# Proteomic Evolution from Acute to Post-COVID-19 Conditions

**DOI:** 10.1021/acs.jproteome.3c00324

**Published:** 2023-12-04

**Authors:** Yassene Mohammed, Karen Tran, Chris Carlsten, Christopher Ryerson, Alyson Wong, Terry Lee, Matthew P. Cheng, Donald C. Vinh, Todd C. Lee, Brent W. Winston, David Sweet, John H. Boyd, Keith R. Walley, Greg Haljan, Allison McGeer, Francois Lamontagne, Robert Fowler, David Maslove, Joel Singer, David M. Patrick, John C. Marshall, Srinivas Murthy, Fagun Jain, Christoph H. Borchers, David R. Goodlett, Adeera Levin, James A. Russell, ARBs CORONA I Consortium

**Affiliations:** † Center for Proteomics and Metabolomics, Leiden University Medical Center, Leiden 2333 ZA, The Netherlands; ‡ UVic-Genome BC Proteomics Centre, University of Victoria, Victoria V8Z 5N3, BC Canada; § Gerald Bronfman Department of Oncology, McGill University, Montreal, QC H3A 0G4, Canada; ∥ Division of General Internal Medicine, Vancouver General Hospital and University of British Columbia, 2775 Laurel St, Vancouver, BC V5Z 1M9, Canada; ⊥ Division of Respiratory Medicine, Vancouver General Hospital, University of British Columbia, Vancouver, BC V5Z 1M9, Canada; # Centre for Health Evaluation and Outcome Science (CHEOS), St. Paul’s Hospital, University of British Columbia, 1081 Burrard Street, Vancouver, BC V6Z 1Y6, Canada; ∇ Division of Infectious Diseases, Department of Medicine, McGill University Health Centre, Montreal, PQ H4A 3J1, Canada; ○ Departments of Critical Care Medicine, Medicine and Biochemistry and Molecular Biology, Foothills Medical Centre and University of Calgary, 1403 29 Street NW, Calgary, Alberta T2N 4N1, Canada; ◆ Division of Critical Care Medicine, Vancouver General Hospital, 2775 Laurel St, Vancouver, BC V5Z 1M9, Canada; ¶ Centre for Heart Lung Innovation, St. Paul’s Hospital, University of British Columbia, 1081 Burrard Street, Vancouver, BC V6Z 1Y6, Canada; †† Division of Critical Care Medicine, St. Paul’s Hospital, University of British Columbia, 1081 Burrard Street, Vancouver, BC V6Z 1Y6, Canada; ‡‡ Department of Medicine, Surrey Memorial Hospital, 13750 96th Avenue, Surrey, BC V3V 1Z2, Canada; §§ Mt. Sinai Hospital and University of Toronto, 600 University Avenue, Toronto, ON M5G 1X5, Canada; ∥∥ University of Sherbrooke, Sherbrooke, PQ J1K 2R1, Canada; ⊥⊥ Sunnybrook Health Sciences Centre, 2075 Bayview Avenue, Toronto, ON M4N 3M5, Canada; ## Department of Critical Care, Kingston General Hospital and Queen’s University, 76 Stuart Street, Kingston, ON K7L 2V7, Canada; ∇∇ British Columbia Centre for Disease Control (BCCDC) and University of British Columbia, 655 West 12th Avenue, Vancouver, BC V5Z 4R4, Canada; ○○ Department of Surgery, St. Michael’s Hospital, 30 Bond Street, Toronto, ON M5B 1W8, Canada; ◆◆ BC Children’s Hospital and University of British Columbia, 4500 Oak Street, Vancouver, BC V6H 3N1, Canada; ¶¶ Black Tusk Research Group, Vancouver, BC V6Z 2C7, Canada; ††† Segal Cancer Proteomics, Centre, Lady Davis Institute for Medical Research, McGill University, Montreal, QC H3T 1E2, Canada; ‡‡‡ Gerald Bronfman Department of Oncology, Jewish General Hospital, Montreal, QC H3T 1E2, Canada; §§§ Division of Experimental Medicine, McGill University, Montreal, QC H3T 1E2, Canada; ∥∥∥ Department of Pathology, McGill University, Montreal, QC H3T 1E2, Canada; ⊥⊥⊥ Division of Nephrology, St. Paul’s Hospital, 1081 Burrard Street, Vancouver, BC V6Z 1Y6, Canada

**Keywords:** COVID-19, post-COVID-19 conditions, restrictive lung disease, targeted quantitative proteomics

## Abstract

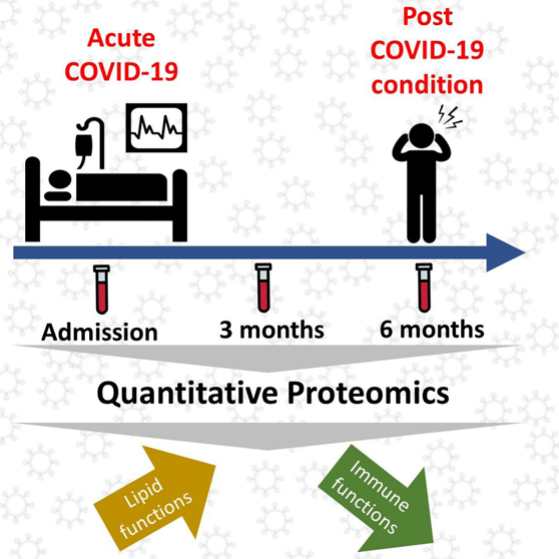

Many COVID-19 survivors have post-COVID-19 conditions, and females are at a higher risk. We sought to determine (1) how protein levels change from acute to post-COVID-19 conditions, (2) whether females have a plasma protein signature different from that of males, and (3) which biological pathways are associated with COVID-19 when compared to restrictive lung disease. We measured protein levels in 74 patients on the day of admission and at 3 and 6 months after diagnosis. We determined protein concentrations by multiple reaction monitoring (MRM) using a panel of 269 heavy-labeled peptides. The predicted forced vital capacity (FVC) and diffusing capacity of the lungs for carbon monoxide (DLCO) were measured by routine pulmonary function testing. Proteins associated with six key lipid-related pathways increased from admission to 3 and 6 months; conversely, proteins related to innate immune responses and vasoconstriction-related proteins decreased. Multiple biological functions were regulated differentially between females and males. Concentrations of eight proteins were associated with FVC, %, and they together had *c*-statistics of 0.751 (CI:0.732–0.779); similarly, concentrations of five proteins had *c*-statistics of 0.707 (CI:0.676–0.737) for DLCO, %. Lipid biology may drive evolution from acute to post-COVID-19 conditions, while activation of innate immunity and vascular regulation pathways decreased over that period. (ProteomeXchange identifiers: PXD041762, PXD029437)

## Introduction

A significant number of individuals who have recovered from COVID-19 continue to experience symptoms even after their acute illness has resolved, a condition commonly referred to as long COVID, long-haul COVID-19, or post-COVID-19 conditions. About 15–35% of acute COVID-19 survivors have post-COVID-19 conditions,^1^ which are characterized by impaired multisystem^2−8^ outcomes. Millions of cases of post-COVID-19 conditions have occurred globally with estimates from the U.K. and USA ranging from 14^9^ to 37%^10^ of COVID-19 cases. The most common symptoms are fatigue, shortness of breath, and cognitive dysfunction.^11^ Post-COVID-19 conditions can occur after hospitalization for acute COVID-19^12−16^ as well as after episodes of community acute COVID-19 that do not require hospitalization.^8,17−19^ Some studies of post-COVID-19 conditions have evaluated only outpatients who developed post-COVID-19 conditions after being released from the hospital, while other studies involved patients hospitalized for acute COVID-19 who later developed post-COVID-19 conditions.^2−7,10,20−25^

The investigation of blood biomarkers that can predict the development and severity of post-COVID-19 conditions remains relatively under-researched. A few promising results have been observed through metabolomics^26^ and transcriptomics^27^ analyses, revealing unique metabolic responses that persist in individuals with post-COVID-19 conditions. Thus, it is logical to develop hypotheses that acute-phase protein levels may differ markedly between acute COVID-19 and healthy controls, which therefore might shed light on possible diagnostic and therapeutic targets for post-COVID-19 conditions.^28−30^ In this context, proteomics stands out as a suitable tool to investigate the dysregulated proteins.

In the current work, we sought to address three questions that remain unresolved in the post-COVID-19 conditions. First, it is unknown whether and to what extent plasma protein levels change from the time of hospital admission to few months later: in our work 3 to 6 months later. Second, females have a higher risk of post-COVID-19 conditions than males,^21^ but the mechanisms behind this observed outcome remain unclear. Third, restrictive lung disease is an important condition leading to dyspnea and fatigue, two of the most common symptoms observed under post-COVID-19 conditions. However, the causes of restrictive lung disease in post-COVID-19 are still uncertain.

The objective of our study was to use quantitative targeted plasma proteomics with an internal standard to quantify plasma proteins and attempt to address these three questions. In addition, our goal was also to perform functional analyses to highlight the molecular functions and biological processes associated with proteins whose abundances change in the three scenarios we studied. To the best of our knowledge, there are no proteomic studies characterizing the latter.

## Methods

### Experimental Design and Rationale

This study was approved by the Providence Health Care and University of British Columbia Human Research Committee (Approval No. H20-00600) and by each of the contributing clinical sites. Anonymized clinical data and use of discarded plasma from clinical blood tests were deemed low risk, and informed consent was deemed not necessary for this research.

ARBs CORONA I is a multicenter cohort of patients in Canada hospitalized for acute COVID-19.^28,31,32^ Inclusion criteria for ARBs CORONA I were patients over 18 years of age who had confirmed SARS-CoV-2 infection (according to a local hospital or provincial laboratories with clinically approved laboratory testing for SARS-CoV-2) who were admitted to the hospital. ARBs I exclusion criteria were acute COVID-19 readmissions, Emergency Department visits only, and admissions in which COVID-19 was not the most responsible diagnosis.

Surviving patients hospitalized for acute COVID-19 at St. Paul’s Hospital and Vancouver General Hospital (Vancouver, Canada) who were in the ARBs CORONA I study were referred to the British Columbia provincial Post-COVID-19 Interdisciplinary Clinical Care Network (PC-ICCN) at 3 and 6 months after hospital admission for acute COVID-19.^33^ Patients in the current study were a subset for whom there was plasma available for research purposes at hospital admission and at 3 and 6 months.

### Baseline Characteristics of COVID-19 Patients

Baseline characteristics included age, sex, and the presence of a previous (i.e., preacute COVID-19) diagnosis of heart failure, hypertension, chronic kidney disease, and diabetes (Table 1). Heart rate, respiratory rate, temperature, blood pressure, arterial oxygen saturation (SaO_2_), serum hemoglobin, creatinine, alanine transaminase (ALT), aspartate transaminase (AST), bilirubin, d-dimer, troponin, platelet count, white blood cell (WBC) count, Glasgow coma score (GCS), and use of vasopressors, invasive ventilation, and renal replacement therapy (RRT) were recorded on the day of admission.

**Table 1:** Baseline Characteristics of Patients Who Were Admitted for Acute COVID-19 and Evaluated at Hospital Admission and at 3 and 6 Months^a^

variable	all (*n* = 74)	male (*n* = 48)	female (*n* = 26)	*P*
sex, *n* (%)				
male	48 (64.9)	48 (100.0)	0 (0.0)	
female	26 (35.1)	0 (0.0)	26 (100.0)	
age, mean (SD)	59.6 (15.7)	61.1 (15.6)	57.0 (15.9)	0.282
comorbidities, *n* (%)				
chronic cardiac disease	16/74 (21.6)	11/48 (22.9)	5/26 (19.2)	0.713
chronic kidney disease	5/74 (6.8)	5/48 (10.4)	0/26 (0.0)	0.155
hypertension	31/73 (42.5)	23/47 (48.9)	8/26 (30.8)	0.133
diabetes	18/74 (24.3)	10/48 (20.8)	8/26 (30.8)	0.342
chronic pulmonary disease (not asthma)	3/73 (4.1)	2/48 (4.2)	1/25 (4.0)	1.000
asthma	9/74 (12.2)	3/48 (6.3)	6/26 (23.1)	0.035
liver disease	2/74 (2.7)	2/48 (4.2)	0/26 (0.0)	0.538
chronic neurological disorder	4/74 (5.4)	1/48 (2.1)	3/26 (11.5)	0.121
malignant neoplasm	3/73 (4.1)	2/47 (4.3)	1/26 (3.8)	1.000
chronic hematologic disease	1/74 (1.4)	1/48 (2.1)	0/26 (0.0)	1.000
AIDS/HIV	3/68 (4.4)	3/45 (6.7)	0/23 (0.0)	0.546
obesity (as defined by clinical staff)	6/74 (8.1)	2/48 (4.2)	4/26 (15.4)	0.176
rheumatologic disorder	9/74 (12.2)	4/48 (8.3)	5/26 (19.2)	0.171
dementia	0/73 (0.0)	0 (0.0)	0 (0.0)	
malnutrition	0/74 (0.0)	0 (0.0)	0 (0.0)	
admitted to ICU on hospital admission day, *n* (%)	12 (16.4)	10 (21.3)	2 (7.7)	0.134
organ support on the admission day				
invasive mechanical ventilation, *n* (%)	6 (8.1)	4 (8.3)	2 (7.7)	1.000
RRT or dialysis, *n* (%)	0 (0.0)	0 (0.0)	0 (0.0)	
vasopressors, *n* (%)	4 (5.4)	3 (6.3)	1 (3.8)	1.000
temperature (°C), mean (SD)*	37.5 (0.9)	37.5 (0.9)	37.3 (0.8)	0.328
heart rate (beats per minute), mean (SD)*	96.2 (19.3)	95.9 (21.6)	96.8 (13.9)	0.855
respiratory rate (breaths per minute), mean (SD)*	24.9 (7.4)	25.3 (8.0)	24.4 (6.3)	0.631
sBP, mean (SD)*	131.1 (19.7)	133.2 (20.9)	126.9 (16.7)	0.193
dBP, mean (SD)*	75.6 (13.2)	76.1 (12.8)	74.7 (14.1)	0.670
oxygen saturation (SaO_2_; %), mean (SD)*	91.3 (5.9)	89.9 (6.2)	93.8 (4.4)	0.006
oxygen status, *n* (%)				0.749
room air	45/72 (62.5)	30/47 (63.8)	15/25 (60.0)	
oxygen therapy	27/72 (37.5)	17/47 (36.2)	10/25 (40.0)	
WBC count (×10^3^/μL), median (IQR)*	6.0 (5.0, 8.2)	6.2 (5.0, 9.0)	5.9 (4.6, 7.5)	0.572
hemoglobin (g/L), median (IQR)*	139 (132, 147)	143 (135, 155)	134 (121, 145)	0.003
creatinine (μmol/L), median (IQR)*	84 (68, 104)	93 (76, 112)	68 (57, 80)	<0.001
potassium (mEq/L), median (IQR)*	3.8 (3.6, 4.1)	3.8 (3.6, 4.0)	3.8 (3.5, 4.2)	0.798
ALT (U/L), median (IQR)	49 (27, 85)	45 (24, 90)	50 (27, 71)	0.756
missing, *n*	7	6	1	
AST (U/L), median (IQR)	66 (37, 97)	70 (34, 96)	62 (40.5, 108)	0.704
missing, *n*	29	19	10	
platelets (×10^9^/L), median (IQR)*	200 (171, 252)	195 (158, 235)	240 (191, 272)	0.004
d-dimer level (ng/mL), median (IQR)	732 (503, 1199)	699 (488, 1073)	896 (538, 1367)	0.464
missing, *n*	28	20	8	
bilirubin (μmol/L), median (IQR)	10.0 (8.0, 12.0)	10.0 (9.0, 12.0)	8.0 (7.0, 11.0)	0.046
missing, *n*	11	7	4	
INR, median (IQR)	1.10 (1.00, 1.20)	1.10 (1.00, 1.20)	1.20 (1.00, 1.20)	0.365
missing, *n*	16	10	6	
troponin (ng/mL), median (IQR)	0.0200 (0.0120, 0.0200)	0.0200 (0.0120, 0.0200)	0.0200 (0.0080, 0.0200)	0.694
missing, *n*	12	8	4	
Glasgow coma scale				0.134
unknown	12	9	3	
13–15	60 (96.8)	39 (100.0)	21 (91.3)	
9–12	2 (3.2)	0 (0.0)	2 (8.7)	
8 or less	0 (0.0)	0 (0.0)	0 (0.0)	
mean arterial pressure (mmHg)	86 (75, 95)	88.5 (77, 99)	81 (75, 89)	0.062
missing, *n*	23	14	9	
FiO_2_ (%), median (IQR)	30 (28, 40)	30 (28, 47.5)	30 (21, 36)	0.326
missing, *n*	12	8	4	
4C mortality score, median (IQR)	7 (5, 10)	8 (5, 11)	5 (4, 8)	0.023
missing, *n*	8	3	5	

a The *p*-value was based on the Chi-square test, Fisher’s exact text, *t*-test, or Wilcoxon rank-sum test as appropriate. * Missing for up to two patients.

Acute COVID-19 severity was based on a modified version of the 4C mortality score^34^ that included in the primary publication age, sex, comorbidities, respiratory rate, SpO2, GCS, and urea and C-reactive protein. The GCS and C-reactive protein were excluded from our calculation of a modified 4C mortality score because data were not consistently captured for the GCS and not at all for C-reactive protein. The definition of comorbidities was based on the predefined items in ARBs CORONA I^31^ instead of those defined by the Charlson comorbidity index as used in the original 4C mortality score. Urea was not captured in our study, and so the serum creatinine level was used to measure renal function at three levels comparable to urea as follows: normal: <110 μ/L; moderate elevation: 110–220 μ/L; and more than moderate elevation: >220 μ/L.

### Post-COVID-19 Condition Outcomes

Patients who were discharged from the hospital after acute COVID-19 were referred to a British Columbia provincial network of five Post-COVID-19 condition clinics.^33^ For the current study, patients were evaluated at St. Paul’s Hospital and Vancouver General Hospital post-COVID-19 condition clinics. We chose two pulmonary function tests used to diagnose restrictive lung disease, percent-predicted vital capacity (FVC%), and percent-predicted diffusing capacity of the lung for carbon monoxide (DLCO%) as outcomes for association of proteomics with post-COVID-19 condition restrictive lung disease. Both respiratory muscle weakness and lung disease can cause post-COVID condition respiratory symptoms, such as breathlessness. We chose FVC% and DLCO% because FVC% can be altered by respiratory muscle weakness or lung disease, whereas DLCO% is altered by lung disease only.

### Measurement of Plasma Protein Levels Using Targeted Quantitative Proteomics

The multiple reaction monitoring (MRM) assays used were developed and validated at the University of Victoria Proteomics Centre, Victoria, BC, Canada,^35−40^ and include stable isotope-labeled internal standard (SIS) peptides for 269 proteins. The MRM assays are characterized according to the Tier 2 Clinical Proteomic Tumor Analysis Consortium (CPTAC) guidelines^41^ and were applied previously for analysis of COVID-19 plasma samples.^28,31,42^ A list of the peptides and proteins is provided in the Supporting Information, Table S1. The concentrations of endogenous proteotypic peptides were determined by comparing their responses in the mass spectrometer to the responses of the heavy-labeled internal standard peptides that had been spiked into the sample, as described below. The order of the sample measurement was randomized using R package Omixer.^43^

The sample digest preparation protocol was developed previously^44^ and was optimized and used in multiple follow-up studies.^36,39,45−50^ In our current work, we used a urea-based protocol in which 10 μL of plasma was diluted with 20 μL of 9 M urea/20 mM dithiothreitol and incubated for 30 min at 37 °C to achieve denaturation and reduction. The samples were alkylated with iodoacetamide (40 mM final concentration) for 30 min at room temperature in the dark, and then, the samples were diluted 10-fold in 100 mM Tris prior to tryptic digestion. Digestion was carried out at 10:1 substrate/enzyme ratio using tosyl phenylalanyl chloromethyl ketone (TPCK)-treated trypsin (Worthington) for 18 h at 37 °C. After digestion, samples were acidified with aqueous 1% formic acid (FA), and a chilled SIS peptide mixture was added. Samples were concentrated via solid-phase extraction (SPE; 10 mg of Oasis HLB cartridges; Waters), using the manufacturer’s recommended protocol. The SPE column was conditioned with 100% methanol (1 mL), followed by washing with 99.9% H_2_O/0.1% FA (1 mL); the sample (diluted to 1 mL using 99.9% H_2_O/0.1% FA) was then loaded onto the column, followed by washing two times with water (1 mL each). Finally, the sample was eluted with 55% acetonitrile (ACN)/0.1% FA (300 μL) and lyophilized to dryness. The dried samples were rehydrated in 0.1% FA to 1 μg/μL for liquid chromatography (LC)/MRM-MS analysis. The samples were separated online with a reversed-phase–ultrahigh-performance liquid chromatography (RP-UHPLC) column (EclipsePlusC18 RRHD 150 mm × 2.1 mm i.d., 1.8 μm particle diameter; Agilent) maintained at 50 °C. Peptide separations were performed at 0.4 mL/min in a 56 min run, via a multistep LC gradient. The solvents were the aqueous mobile phase (solvent A), which contained 0.1% formic acid in LC-MS-grade water, and the organic mobile phase (solvent B), which contained 0.1% formic acid in LC-MS-grade acetonitrile. The exact gradient was as follows (time point in minutes, solution B%): 0 min, 2%; 2 min, 7%; 50 min, 30%; 53 min, 45%, 53.5 min, 80%; 55.5 min, 80%; and 56 min, 2%. A postcolumn equilibration of 4 min was used after each sample analysis. The LC system was interfaced to a triple-quadrupole mass spectrometer (Agilent 6490) via a standard-flow electrospray ionization (ESI) source, operated in positive ion mode. The MRM acquisition parameters employed for the quantitation were as follows: 3500 V capillary voltage, 300 V nozzle voltage, 11 L/min sheath gas flow at a temperature of 250 °C, 15 L/min drying gas flow at a temperature of 150 °C, 30 psi nebulizer gas pressure, 380 V fragmentor voltage, 5 V cell accelerator potential, and unit mass resolution in the first and third quadrupoles. The peptide-specific collision energy (CE) values for optimal peptide collision-induced dissociation had previously been determined experimentally. The exact CE value for each peptide is available from PeptideTracker^38^ (http://peptidetracker.proteincentre.com/).

### Previous Datasets for Additional Comparison

In the current work, we included the proteomic plasma profiles of healthy controls from our previous work^28^ as a reference for any innate differences in the plasma protein levels between females and males. The samples were analyzed as part of the previous work using the same MRM analytical method and protein panel. Six healthy female individuals aged 19–50 years of age (mean: 41.5) and eight healthy males aged 18–57 years (mean: 34.5) of the same background were included.

### Sensitivity Analyses for Pre-existing Lung Disease

Pre-existing lung disease could affect the interpretation of the association between proteomic analyses and pulmonary function (FVC, %, and DLCO, %). We therefore performed four different exclusion/inclusion analyses and compared the results based on change in c-statistics when evaluating associations of protein levels with FVC, %, or DLCO, %, i.e., change in the area under the receiver operating curve in a cross-validated regression model for discrimination. The model uses proteins differentiated in their abundance to discriminate between patients with FVC, %, or DLCO, % values above or below 80% because 80% is the usual threshold of normal. The four analyses were (1) results from all patients, (2) analysis excluding the nine patients who had asthma, (3) analysis that excluded only the three patients who had chronic pulmonary disease, and (4) analysis in which all 12 of the aforementioned patients with pre-existing pulmonary condition were excluded.

### Data Processing

Skyline was used to inspect the peptide response peaks and to ensure accurate selection, retention time, integration, and uniformity of peak shape for the endogenous and internal standard peptide signals.^51^ For each peptide, the relative peak area ratio of endogenous to heavy-labeled internal standard peptide was calculated. This ratio and the known concentration of the internal standard peptide were used to calculate the concentration of the endogenous peptide in the sample by comparison to a standard curve generated in the pooled sample. The criteria used for the standard curve regression analysis were 1/*x*^2^ regression weighting, <15% deviation in a given level’s precision and accuracy for each concentration level, and 20% at the lower limit of quantification.

### Statistical Analyses

Protein concentrations are reported in fmol/μL; other clinical descriptive and data are described as number (percent), mean ± standard deviation, or median (interquartile range), as appropriate. The unsupervised cluster analysis was performed using the protein concentrations determined. We used the complete distance to perform the clustering on the scaled and centered concentration values. Visualization of the data using heatmaps was performed after centering and scaling of the determined protein concentrations. Differences between female and male healthy controls were tested using the Wilcoxon rank-sum test. *p*-values were adjusted with the Benjamini–Hochberg method to account for multiple testing. Fold changes were calculated on a base-2 logarithmic scale after dividing the individual protein concentrations by the corresponding reference abundance of the protein. Statistical significance was defined by a *p*-value less than 0.05 after correction for multiple testing. Significant fold change was set to detect a 25% increase or decrease in protein abundance, which is based on the variation in our overall MRM experiment and is determined from QC samples that have been analyzed multiple times in prior studies and in the current work. The value reflects that approximately 70% of the quantified proteins have CVs less than 25%. The baseline in our longitudinal comparisons was the corresponding patient protein abundance at admission. Partitional time series clustering with the Manhattan distance was used to identify protein profile clusters over time. For the longitudinal analysis and differences between female and male patients, *p*-values were asserted from two ways analysis of variance (ANOVA) and adjusted with the Benjamini–Hochberg method to account for multiple testing. Significantly differentiated proteins as well as proteins belonged to identified time series clusters considered in functional analyses, which were performed using Cytoscape^52^ and the Cytoscape plugin GeneMANIA^53^ to understand the pathways that were significantly perturbed in the groups. Top pathways that were specific to each comparison were selected for further analysis as well as static figures reported here, while a link to the full interactive visualization is provided in the results and the Supporting Information Materials. Differences in protein abundances between groups at admission, specifically between patients with forced vital capacity (FVC, %) and lung capacity for carbon monoxide (DLCO, %) percentage values above and below the threshold of 80%, were tested using the Wilcoxon rank-sum test. Prediction was performed using regression analysis on proteins with a *p*-value less than 0.05 and a log fold change of 0.3. Validation was performed using cross-validation to estimate c-statistics and associated confidence intervals and was performed using 30/70% training and testing sets that were drawn randomly from the samples and repeated 100 times. All data analysis and visualization were performed using R (version 4.2.1),^54^ Cytoscape (version 3.8.2), and its GeneMANIA (version 3.5.2) plugin.^52,53^

## Results

### Sample Cohort

The 74 patients with COVID-19 were admitted to the hospital for acute COVID-19 between March 5, 2020 and April 1, 2021 (Table 1). Patients had a mean age of 59.6 years with a standard deviation of 16 years. Out of all patients, 48 were males and 26 were females. Most common comorbidities were hypertension (42.5%), diabetes (24%), and chronic cardiac disease (22%). Only nine patients (12%) had asthma and three (4%) had chronic pulmonary disease. Plasma sampling occurred on admission and again at 3 and 6 months. The patients in the current study were similar in sex distribution to the overall British Columbia Post-COVID-19—Interdisciplinary Clinical Care Network^55^ (Table S2).

### MRM-Based Proteomics and Plasma Protein Signatures

We determined protein concentrations in blood plasma samples obtained from acute COVID-19 patients at admission and after 3 and 6 months using MRM with internal standards. The approach we used is well-suited for studies like ours that are longitudinal, multicenter studies because it references measured peptide intensities to the signals of spiked-in internal standards, allowing the absolute quantification of target proteins via their peptide surrogates. In previous work, it has been shown that plasma proteomics can identify up to 900 proteins;^56^ however, quantification also relies on additional quality criteria, namely that acceptable determined concentrations had to be within the dynamic range of a standard curve that is generated as part of the experiment. We used a quantitative proteomic panel for 269 plasma proteins that we had thoroughly validated in previous studies.^39,57,58^ The panel included internal standards for all proteins (Table S1) and has been previously characterized as showing good reproducibility.^58^ The panel typically quantifies 160–175 proteins depending on the quality of the plasma samples and the anticoagulant used.^58^ In the current study, we were able to detect 192 proteins, of which 172 were quantified; no imputation was performed, and we used a nonparametric test for all comparisons. We considered a protein to be quantifiable if its determined concentrations in 90% of the samples were above the lower limit of quantification (LLOQ), which was determined using regression analysis and a standard curve generated in the same experiment.^36^

**Figure 1:**
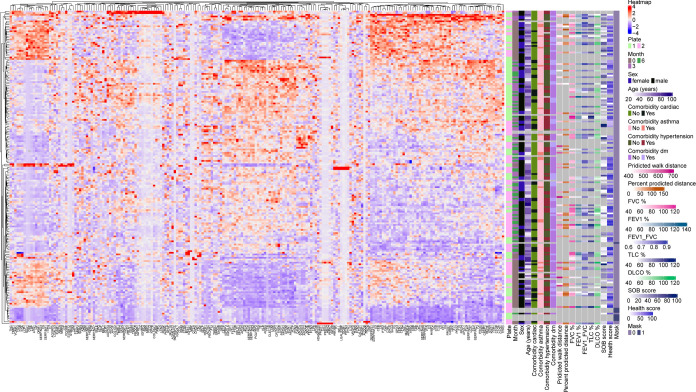
Heatmap of the plasma proteomic expression in patients who were admitted for acute COVID-19 and were evaluated at hospital admission and at 3 and 6 months.

A heatmap of proteins at hospital admission and 3 and 6 months is shown in Figure 1. The horizontal hierarchical clustering divided patients into several unique and distinct subgroups based on their protein signatures linked in clusters. The clustering guiding the orders did not show any association with the measurement batch.

### Longitudinal Analyses of Proteomics from Hospital Admission to Six Months

**Figure 2:**
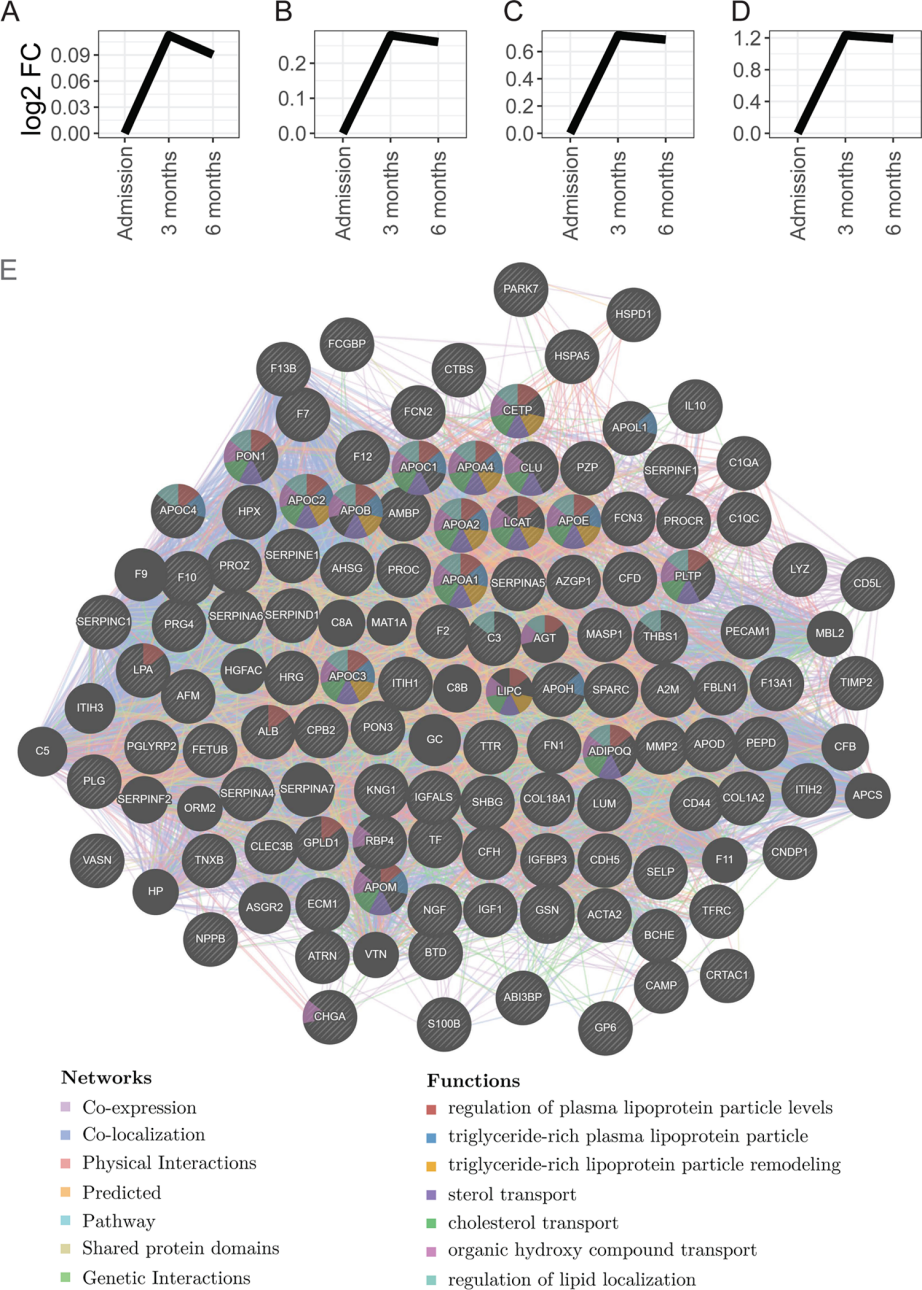
Functional analysis of the proteins showed an increase in abundance from admission for acute COVID-19 to 3 and 6 months. (A–D) Four trends of protein clusters that had increased in their abundances from admission to 3 and 6 months. The trends in abundances are shown in relation to the concentration measured at admission and depicted in the plots in the Log_2_ fold change. The four trends identify proteins with different levels of fold change increase as shown on the vertical axis. The lists of proteins in each cluster are provided in the Supporting Information in Figure S1. E shows the results of the functional analysis as the association network of the proteins. Nodes are the proteins, edges are associations between the proteins colored according to the type (coexpression, colocalization, genetic interactions, pathway, physical interactions, predicted, or shared protein domains), and functions are mapped to the node in color as in the legend. The dynamic network can be accessed via the following link https://tinyurl.com/56wbzzyv (the full link is provided in the Supporting Information links).

**Figure 3:**
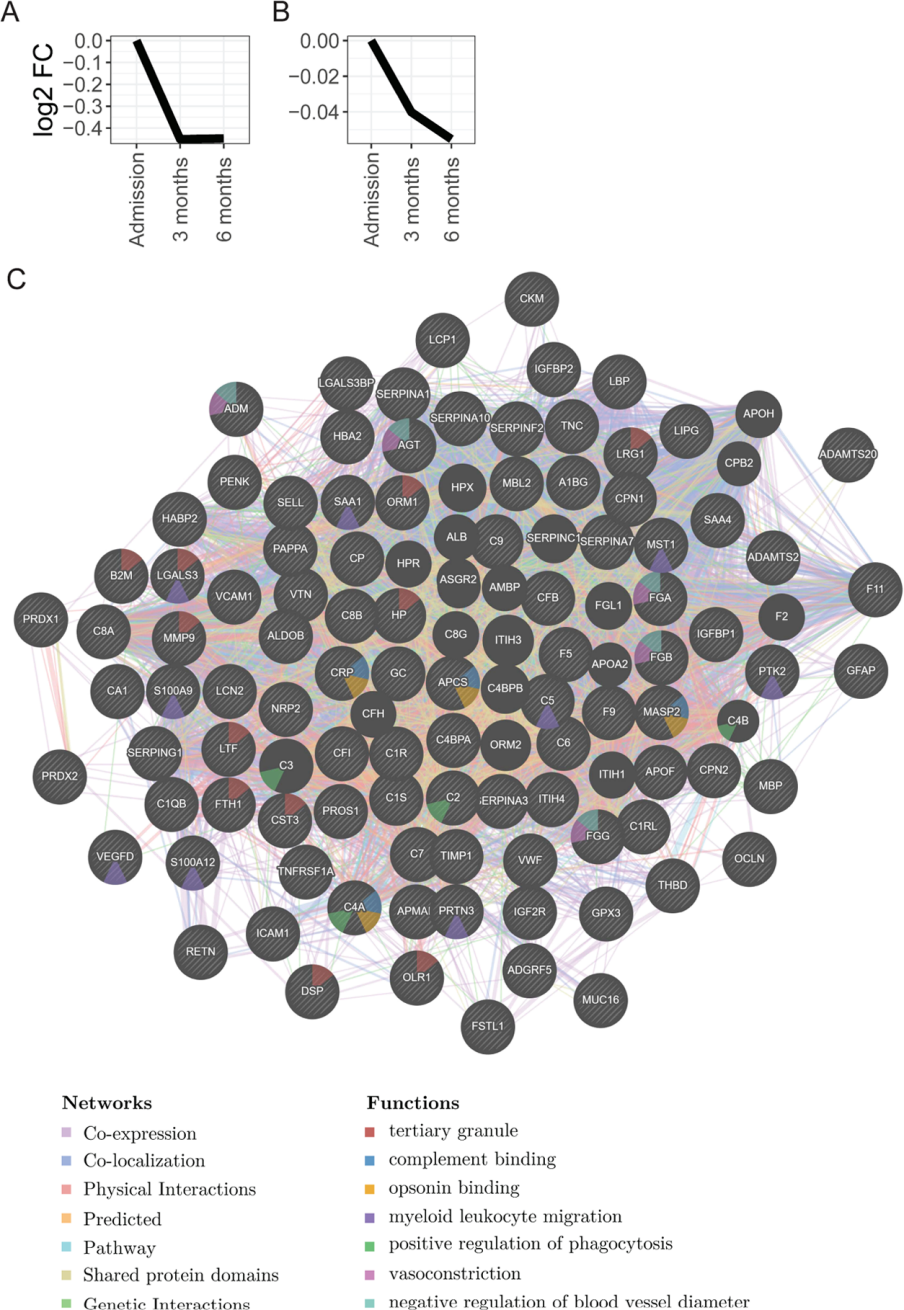
Functional analysis of the proteins showed a decrease in abundance from admission for acute COVID-19 to 3 and 6 months. (A, B) Trends of proteins with decreased abundance from admission to 3 and 6 months. The changes in protein abundances are relative to the protein concentrations at admission and are represented as the Log_2_ fold change. The two trends identify proteins with different levels of decreased fold change as shown on the vertical axis. The lists of proteins in each cluster are provided in Supporting Information Figure S1. C shows the functional analysis as an association network of the proteins. Nodes represent the proteins, edges are associations between the proteins colored according to the type (coexpression, colocalization, genetic interactions, pathway, physical interactions, predicted, or shared protein domains), and the functions are mapped to the node in color as in the legend. The dynamic network can be reviewed online via the following link https://tinyurl.com/2s3jdesc (the full link is provided in the Supporting Information links).

Only a few proteins showed significant changes in their determined abundances as acute COVID-19 evolved into post-COVID-19 conditions. We did, however, identify two major trajectories of proteins whose concentrations changed significantly: low-to-high and high-to-low transitions of protein concentration from admission to 3 and 6 months. There were six unique clusters of proteins; four of these increased from hospital admission to 3 and 6 months, while two decreased during this time (Figures 2 and 3). In the clusters that increased from hospital admission to 3 and 6 months, key lipid-related pathways increased, including regulation of plasma lipoprotein particle levels, triglyceride-rich plasma lipoprotein particles, triglyceride-rich lipoprotein particle remodeling, sterol transport, cholesterol transport, and regulation of lipid localization (Figure 2). Proteins and functions that decreased from admission to 3 and 6 months included many immune responses, specifically proteins related to leukocytes, structural and binding properties including tertiary granules, complement binding, opsonin binding, myeloid leukocyte migration, and positive regulation of phagocytosis (Figure 3). There were decreases in the concentrations of proteins related to vasoconstriction and negative regulation of the blood vessel diameter.

### Proteomic Signature in Post-COVID-19 Conditions: Associations with Sex

**Figure 4:**
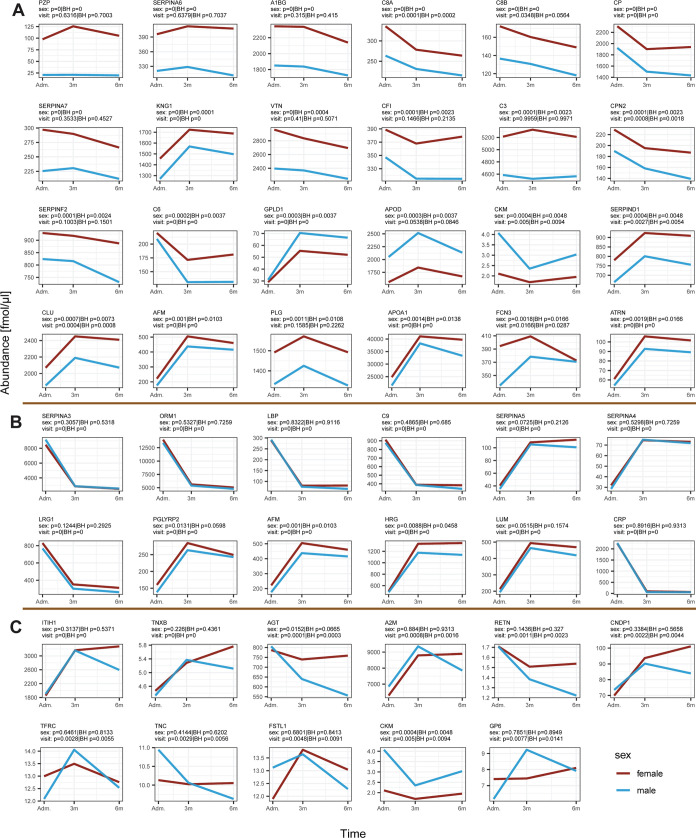
Three main patterns of proteins differed according to sex in post-COVID-19 patients. Each plot represents abundance levels of proteins at admission to the hospital for acute COVID-19 (Adm.) as well as at 3 months (3m) and 6 months (6m) after hospitalization. The vertical *y*-axis shows protein concentration in fmol/μL. Figure 4A shows proteins that had parallel longitudinal trends but different protein abundances. Figure 4B shows proteins with very similar longitudinal trends and abundances. Figure 4C shows proteins that had a pattern of inconsistent trends between males and females.

Although many proteins differed in abundance between females and males, there were three clear patterns of longitudinal trends and abundance levels. First, several proteins had similar longitudinal trends but different abundance levels (Figure 4A); second, some proteins had similar longitudinal trends and abundance levels (Figure 4B); third, some proteins had different trends and different levels between females and males (Figure 4C).

**Figure 5:**
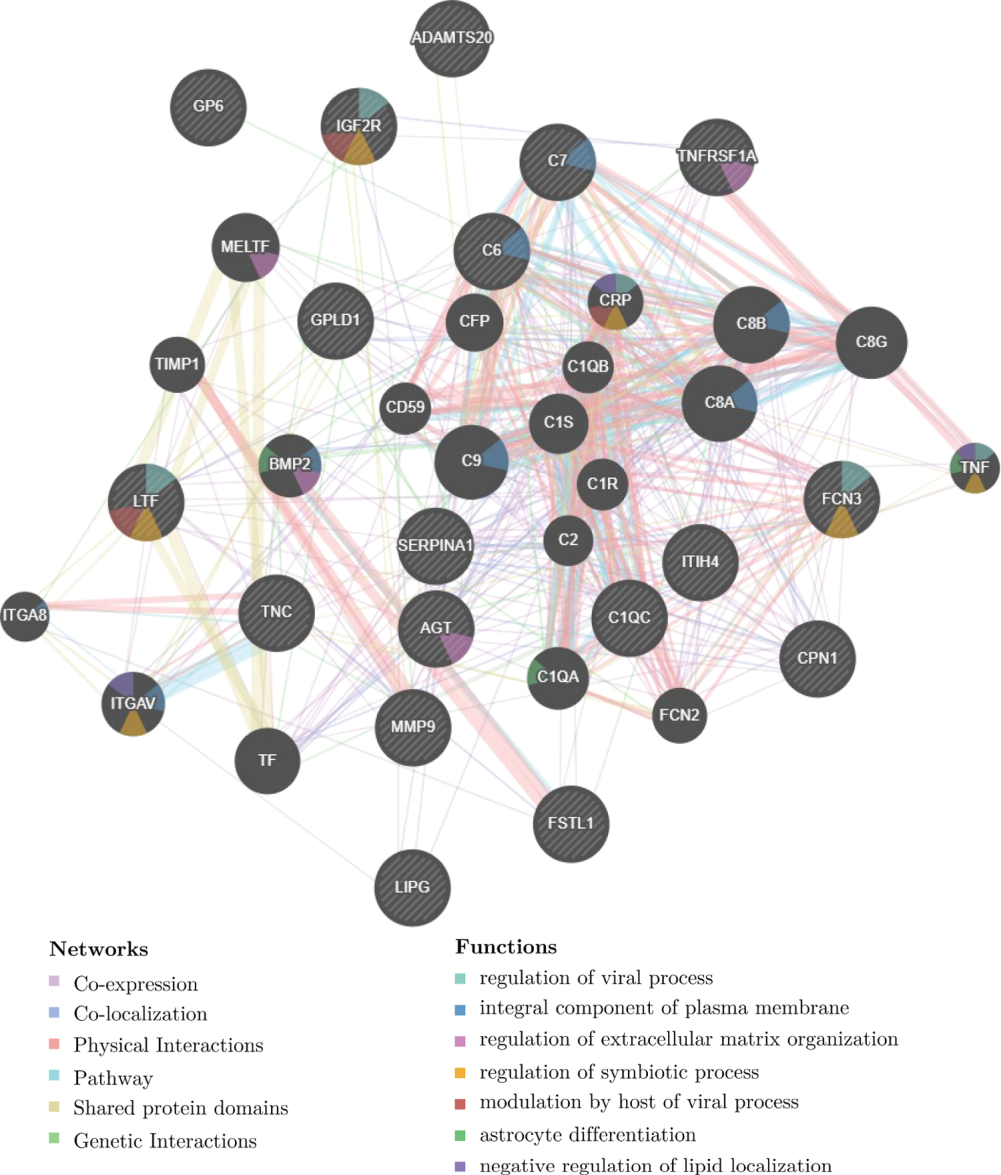
Functional analysis of the proteins associated with the longitudinal sex difference shown in Figure 2A of patients who were admitted for acute COVID-19 and were evaluated at 3 and 6 months.

The proteins that were different between females and males at 3 and 6 months were related to regulation of viral processes, components of plasma membranes, extracellular matrix organization, symbiotic processes, astrocyte differentiation, and negative regulation of lipid localization (Figures 5). To verify that these differences were due to post-COVID-19 conditions rather than just sexual differences, we compared the proteins that exhibited differential regulation based on sex in the current post-COVID-19 cohort against those observed in healthy control individuals. In our previous work, we studied patients hospitalized for acute COVID-19 with plasma specimens available for the first 2 weeks of hospitalization and compared their plasma profile with those of healthy individuals.^28^ The healthy individuals were on average younger than the COVID-19 patients in our current study as well as in our previous cohort. In the previous work, we extensively analyzed the age signature of the plasma protein profile and concluded that the difference in plasma proteomics was not due to age.^28^ The sample size of the healthy individuals, six females and eight males, is small compared to the patient cohort, but the goal of including these samples was to have an indication about the proteins showing sexual dimorphism in healthy controls and to verify whether any such dimorphism is indeed present in COVID-19 patients. For this goal, we decided to consider only a limited number of individuals with similar demographic backgrounds.

In the current work, 12 proteins differed between females and males in the healthy controls before multiple testing correction, and only one protein differed after multiple testing correction. Of the 19 proteins that differed according to sex in post-COVID condition patients, only phosphatidylinositol glycan-specific phospholipase D (GPLD1) was lower in females than in males in post-COVID condition patients as well as in healthy controls. GPLD1 is a secreted enzyme associated with hydrolase and lipid metabolism. The concentration of GPLD1 in male and female patients was very similar at admission and increased over 6 months to reach values similar to those of healthy controls. Removing GPLD1 from the functional analysis did not affect the enriched functions and pathways according to sex in post-COVID condition patients. Protein levels were measured longitudinally over six months in post-COVID condition patients but at only one time in the healthy controls because we assumed that protein levels were similar over time in healthy controls.

### Post-COVID-19 Proteomics and Pulmonary Function Indicating Restrictive Lung Disease

**Figure 6:**
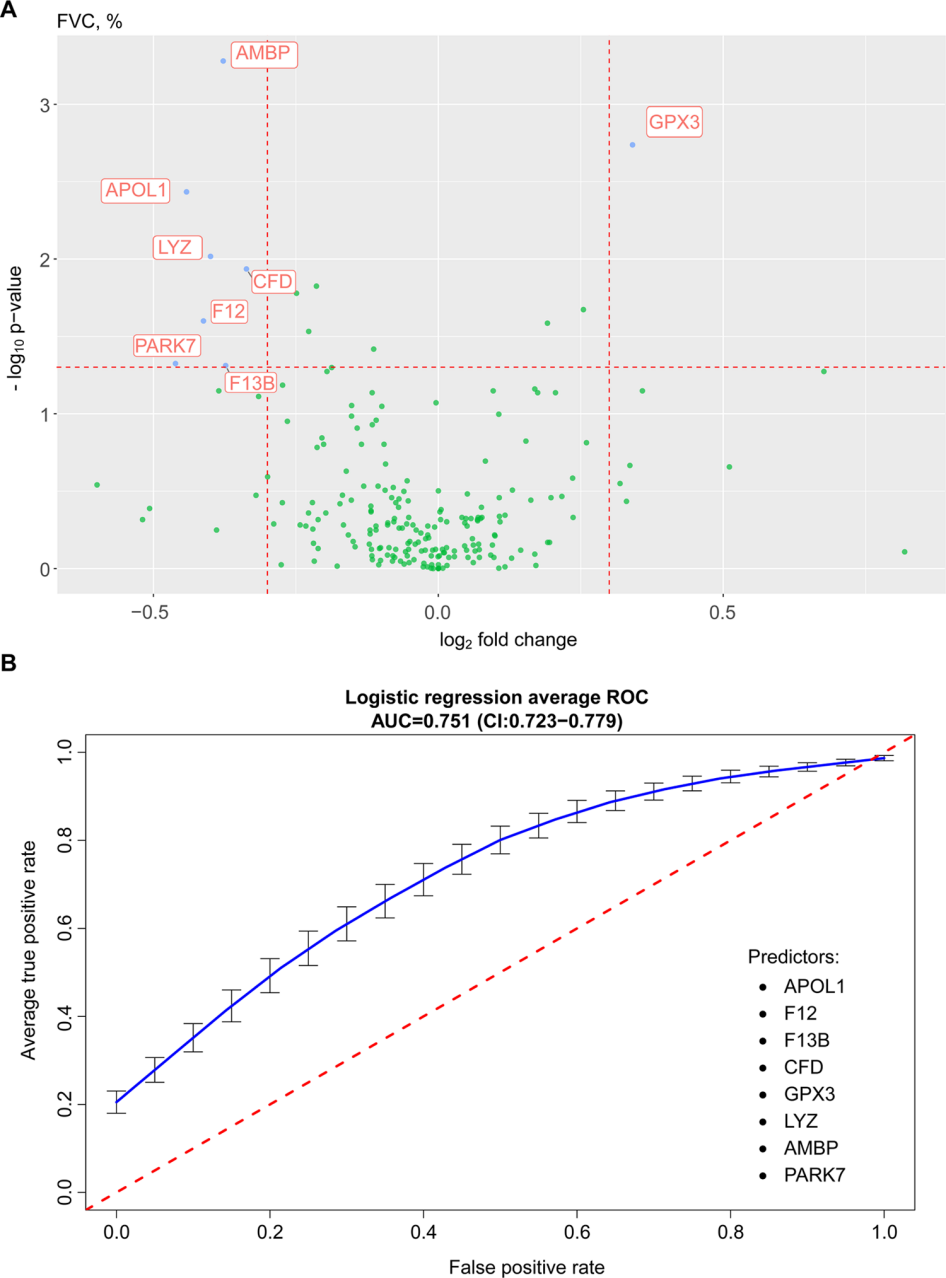
Volcano plot (A) and area under the receiver operating characteristic curve (B) for proteins versus forced vital capacity % predicted of patients who were admitted for acute COVID-19 and who were evaluated at 3 and 6 months. Logistic regression receiver operating characteristic curve showed c-statistics of 75.1% for prediction of forced vital capacity % predicted based on the abundance of eight proteins.

**Figure 7:**
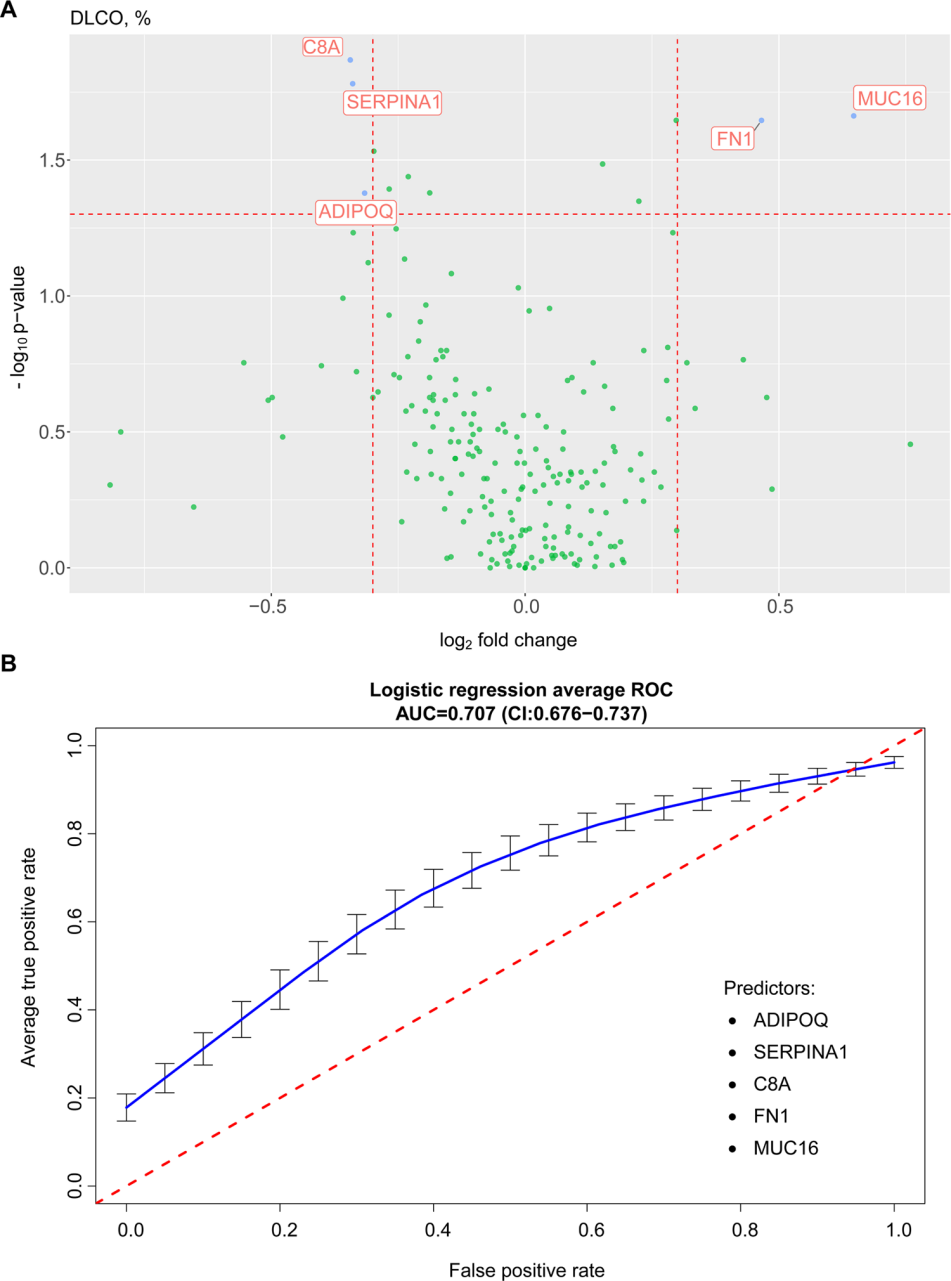
Volcano plot (A) and area under the receiver operating characteristic curve (B) for proteins versus diffusing lung capacity for carbon monoxide % predicted (DLCO, %) of patients who were admitted for acute COVID-19 and who were evaluated at 3 and 6 months. Logistic regression receiver operating characteristic curve showed c-statistics of 70.7% for prediction of diffusing lung capacity for carbon monoxide % predicted based on the abundance of five.

Our proteomic results were associated with the severity of post-COVID-19 restrictive lung disease as measured by FVC, %, and DLCO, % (Figures 6 and 7). The protein apolipoprotein L1 (APOL1), coagulation factors 12 (F12) and 13B (F13B), complement factor D (CFD), glutathione peroxidase 3 (GPX3), lysozyme (LYZ), α-1-microglobulin/bikunin precursor (AMBP), and Parkinsonism-associated deglycase 7 (PARK7) were associated with FVC, %. These eight proteins showed an area under the receiver operating curve, AUC, of 0.751 (CI:0.732–0.779) in a cross-validated regression model for predicting FVC, % (Figure 6). Functional analysis on these proteins indicated activation of the complement system and association with plasma lipoprotein particles. Proteins that were associated with DLCO, %, prediction were adiponectin (ADIPOQ), α-antitrypsin (SERPINA1), complement component 8A (C8a), fibronectin (FN1), and mucin 16 (MUC16). The five-protein panel had an AUC of 0.707 (CI:0.676–0.737) (Figure 7). Interestingly, in the functional analysis, these proteins were associated with the regulation of the humoral immune system as well as the pore complex, but no association to lipoprotein particles was present.

In the sensitivity analyses in which we excluded the nine patients who had asthma and three patients who had chronic pulmonary disease, there were slight differences in the proteins associated with FVC, %, and DLCO, %. Removing either or both of these subgroups affected the outcome of the analysis slightly with AUC in the case of FVC, %, model changing from 0.751 (CI:0.732–0.779) to 0.700 (CI:0.669–0.731), 0.755 (CI:0.728–0.783), and 0.708 (CI:0.676–0.740) when excluding the nine asthma patients, the three chronic pulmonary disease patients, and all 12 together, respectively. In the case of DLCO, %, model, the AUC changed from 0.707 (CI:0.676–0.737) to 0.750 (CI:0.72–0.78), 0.66 (CI:0.627–0.693), and 0.737 (CI:0.706–0.768) when excluding the nine asthma patients, the three chronic pulmonary disease patients, and all 12 together, respectively.

## Discussion

The current study extends our prior exploration of the proteomics of acute COVID-19^28^ and several other proteomic studies of acute COVID-19 that have identified potential acute COVID-19 therapeutic targets^26,29,30^ by investigating plasma protein profiles in post-COVID-19 conditions. In the current work, we investigated post-COVID-19 conditions and found several lipid-related protein pathways that differed from admission to 3 and 6 months, a few protein signals that differed between females versus males, and several plausibly pathogenic proteins that were associated with worse restrictive lung disease.

The main novel features of our work are as follows; first, we show that several proteins differ in longitudinal trends and levels from hospital admission to 3 and 6 months between females and males; second, we discovered completely novel protein signatures with a high area under the concentration curve that are significantly associated with objective pulmonary function, evidence of restrictive lung disease; and third, we show for the first time that most of the pathways that increase from admission to 3 and 6 months are pathways that regulate lipid levels and lipid function.

### Functions Activated in the Initial Days of Acute COVID-19

In our previous work, protein levels, pathways, and associated functions differed between healthy controls and patients hospitalized for acute COVID-19^28^ that reflected acute inflammatory response, complement activation, and protein activation cascade. Longitudinal analysis over 14 days of hospitalization showed increased lipid-associated functions, a rapid decrease and rebound of complement activation, humoral immune response, and acute inflammatory response-related proteins, and constant fluctuations in the regulation of smooth muscle cell proliferation, secretory mechanisms, and platelet degranulation. In the current work, in which we follow acute COVID-19 patients over a longer period of 6 months, two observations are noteworthy. First, the activation of the immune response resolves as the patient transitions from the acute phase of the infection, which is expected. Second and of particular interest, the initial disruptions in lipid homeostasis observed in the first 2 weeks continued in the post-COVID-19 conditions. Further elaboration on this is provided in the following sections.

### Lipid Homeostasis and Post-COVID-19

Multiple proteins associated with several lipid-related pathways increased in abundance from acute to post-COVID-19 conditions, suggesting that lipid dysregulation may contribute to the development of post-COVID-19 conditions. Lipid localization and transport proteins increased significantly, specifically regulation of plasma lipoprotein particle levels, triglyceride-rich plasma lipoprotein particle and triglyceride-rich lipoprotein particle remodeling, sterol transport, cholesterol transport, and regulation of lipid localization. These findings regarding proteins associated with lipid metabolism are consistent with earlier studies by others, which indicated a unique post-COVID-19 condition lipidome signature.^59,60^

Our finding of lipid pathway dysregulation in COVID-19 is aligned with a recent randomized controlled trial of proprotein convertase subtilisin/kexin type 9 (PCSK9) inhibition using the monoclonal antibody Evolucumab.^61^ Evolocumab treatment decreased inflammation and mortality of patients with acute COVID-19.^61^ PCSK9 is a critical regulator of lipid levels because PCSK9 regulates low-density lipoprotein receptor recycling, thereby modulating LDL^62^ levels. PCSK9 inhibition also increases lipopolysaccharide^63,64^ and lipotechoic acid^65^ clearance in Gram-negative and Gram-positive bacterial sepsis and improves outcomes of sepsis animal models. Our proteomic findings and this positive trial of PCSK9 inhibition in human acute COVID-19 suggest the hypothesis that PCSK9 inhibition could decrease the severity of post-COVID-19 conditions.

### Molecular Functions Decreased in Post-COVID-19

A few molecular functions decreased significantly from admission to 3 and 6 months. These included the innate immune response (tertiary granules, complement binding, opsonin binding, myeloid leukocyte migration, and positive regulation of phagocytosis) and vascular vasomotion (vasoconstriction and negative regulation of blood vessel diameter). This indicates that the activation of the innate immune response and the vasoactivity are characteristics of the acute phase, and they dissipate upon recovery from the infection.

### Difference between Female and Male Patients in Post-COVID-19

In an earlier proteomic study of post-COVID-19 conditions, Li and colleagues^66^ found differences in the extracellular matrix, immune response, hemostasis pathways, lipid metabolism, immune response, and pulmonary fibrosis-related proteins in COVID-19 survivors at 6 months. Zoodsma and colleagues^67^ identified several inflammatory protein pathways that were elevated (mediators of the tumor necrosis (TNF)-α and transforming growth factor (TGF)-ß signaling pathways) in the transition from acute COVID-19 to post-COVID-19 conditions several weeks later. To the best of our knowledge, our work is the first study to use proteomics to identify differences between females and males in the evolution from acute to post-COVID-19 conditions. We identified functions related to the regulation of viral processes, components of plasma membranes, extracellular matrix organization, symbiotic processes, astrocyte differentiation, and negative regulation of lipid localization. These differences in protein regulation could explain in part why females have a higher risk of developing more severe post-COVID-19 conditions.^68^ This is consistent with previous observations that there is a post-COVID-19 condition signature on the lipidome level.^59,60^

Females who had acute COVID-19 more frequently have decreased DLCO, %, and 6 min walk test at follow-up; being female is an independent risk factor for decreased DLCO, %, and 6 min walk test.^69^ Differences in plasma proteomics of females correlate with the observation that females have less improvement in pulmonary function with exercise therapy in post-COVID-19 conditions.^70^

### Role of Restrictive Lung Disease and Impaired Pulmonary Function

Restrictive lung disease and impaired pulmonary function are important components of post-COVID-19 conditions that correlate with breathlessness and impaired 6 min walk test.^71−73^ We found that several plausible protein pathways were associated with worse quantitative diagnostic markers (FVC, % predicted, and DLCO, % predicted) of restrictive lung disease. The main results did not change when we did sensitivity analyses and excluded the patients who had pre-existing asthma and COPD.

Different proteins and pathways were associated with FVC, %, predicted, and DLCO, %, predicted, suggesting that different proteins and pathways are associated with respiratory muscle weakness versus interstitial lung disease. In general, functional analysis of proteins associated with FVC, %, predicted indicated activation of the complement system and association with plasma lipoprotein particles. Proteins that were associated with DLCO, %, predicted were proteins associated with the humoral immune system and pore complex regulation but with no lipoprotein associations.

### Forced Vital Capacity and Post-COVID-19

Several functions and pathways were associated with FVC, %, predicted, including apolipoprotein-related pathways, coagulation factors, complement components, peroxidation, and lysozyme. Apolipoprotein L1 (apoL1) that was decreased in patients with reduced FVC, %, predicted, is a minor component of HDL and circulates with HDL3, and both interferon-γ and TNF-α increase apolipoprotein L1. ApoL1 is associated with focal glomerulosclerosis and HIV-associated nephropathy,^74^ and renal dysfunction also frequently complicates acute^75^ and post-COVID-19 conditions.^33^ Abundances of coagulation factors XII and XIIIB (F12, F13B) were also differentiated; F12 increases the generation of angiotensin and bradykinin, both of which are central to the pathogenesis of acute COVID-19.^76−78^ Complement activation is also fundamental to the pathogenesis of acute COVID-19.^79^ Complement factor D (CFD) cleaves complement factor B,^80,81^ and deficiency of CFD is associated with the increased risk of Neisseria infection. Glutathione peroxidase 3 (GPX3) detoxifies hydrogen peroxide; hydrogen peroxide causes pulmonary epithelial injury^82^ in pneumococcal pneumonia^83^ and acute respiratory distress syndrome,^84^ a common complication of acute COVID-19. Lysozyme (LYZ) is an innate immunity glycoside hydrolase with potent antimicrobial activity.^85^ Although lysozyme protects against corneal SARS-CoV-2,^86^ its role in systemic COVID-19 remains unknown. α-1-Microglobulin/bikunin precursor (AMBP), a plasma glycoprotein, is catalyzed to form α-1-macroglobulin that regulates the inflammatory response but has not, to date, been reported to modulate inflammation in COVID-19. Parkinson disease protein 7 (PARK7) is a sensor of oxidative stress that may be relevant in COVID-19 by protecting against neuron cell death.

### Diffusing Capacity of the Lungs for Carbon Monoxide and Post-COVID-19

Five proteins with known lung injury or acute COVID-19 injury mechanisms were significantly associated with DLCO, %, in post-COVID-19 conditions. Adiponectin (ADIPOQ), an adipose tissue-derived protein hormone, regulates glucose and fatty acid oxidation; low adiponectin to leptin levels occur in acute COVID-19 pneumonia^87^ and may be important in post-COVID-19 condition restrictive lung disease. α-Antitrypsin (SERPINA1) is a protease inhibitor that protects against COPD emphysema; α-antitrypsin deficiency causes emphysema, by protecting against neutrophil elastase-induced lung injury. α-Antitrypsin treatment in acute COVID-19 mitigated inflammation and improved lung function.^88^ Complement component 8A (C8A), a terminal complement pathway component, interacts with coagulation to cause lung injury in COVID-19. Fibronectin (FN1) is a coagulation component that marks illness severity of acute COVID-19^89^ that could contribute to post-COVID-19 condition restrictive lung disease. Mucin16 (MUC16) is a protective component of pulmonary epithelial cells, identified in a multimucin signature for acute COVID-19.^90^

A different set of proteins was associated with FVC, %, than with DLCO, %, for reasons that are still unclear. Respiratory muscle weakness causes breathlessness without abnormal FVC, %, in post-COVID-19 conditions^71,72^ and is best detected by measuring the maximum inspiratory force,^91^ but unfortunately, we did not measure that. FVC, %, decreased because of respiratory muscle weakness, interstitial lung disease, or both. In contrast, DLCO, %, decreased because of interstitial lung disease not respiratory muscle weakness. Severe acute COVID-19 is associated with more breathlessness and a lower DLCO, %, value in post-COVID-19 conditions.^73,92^ Perhaps, the two different causes of restrictive lung disease in post-COVID-19 conditions explain why we found different proteins associated with FVC, %, than with DLCO, %; the dysregulation of different proteins may cause respiratory muscle weakness versus the interstitial lung disease in post-COVID-19 conditions.

### Strengths and Limitations of Our Work

The strengths of our study include the longitudinal design of a sample of acute COVID-19-hospitalized patients in whom we were able to make repeated measurements of plasma protein levels at the baseline and at 3 and 6 months, a female versus male comparison, and the associations of protein pathways with quantitative markers of restrictive lung disease under post-COVID-19 conditions. Other strengths were the multicenter design enhancing generalizability, the evaluation of differences in proteins by sex in healthy controls, and the restrictive lung disease sensitivity analysis, in which we removed patients who had pre-existing lung disease. In our longitudinal analysis, we referenced each protein to its own baseline protein level.

The limitations of our study were that we included samples that were collected at hospital admission so we cannot infer possible COVID-19 effects on plasma protein levels at earlier, prehospital admission times. Blood was processed within a 4 h window from collection and that may be viewed as a limitation. Patients may not be representative of all post-COVID-19 condition patients but were representative of the overall British Columbia post-COVID-19—Interdisciplinary Clinical Care Network.^55^ Also, there is the possibility of false negative, i.e., proteoforms with very low abundance, transient, or not blood-based that were not captured in this experimental design. Finally, pre-existing pulmonary disease could contribute to FVC, %, and DLCO, %, findings, but we suggest that the contribution was small because sensitivity analyses with these patients removed did not change our overall findings.

## Conclusions

Lipid biology appears central to evolution from acute to post-COVID-19 conditions because at least six lipid regulation-related pathways increased from hospital admission to 3 and 6 months. In contrast, innate immunity and vascular regulation pathways decreased over that period. The female propensity for post-COVID-19 conditions (compared to males) may be due to differential expression of several protein pathways that regulate viral processes, plasma membranes, extracellular matrix, symbiotic processes, astrocyte differentiation, and lipid localization. Plausible protein pathways, which could be potential drug targets, were associated with more severe worse restrictive lung disease.

## Data Availability

The mass spectrometry proteomics raw data as well as the associated skyline documents have been deposited to the ProteomeXchange Consortium via the PRIDE partner repository with the dataset identifier: PXD041762. Additional data from our previous work^28^ and used for comparison in the current work are available from PRIDE with the identifier: PXD029437.

## Supporting Information Available

The following Supporting Information is available free of charge at ACS Web site https://eur03.safelinks.protection.outlook.com/?url=http%3A%2F%2Fpubs.acs.org%2F&data=05%7C01%7Cy.mohammed%40lumc.nl%7C1d7267b5bd214acfe69608db84449464%7Cc4048c4fdd544cbd80495457aacd2fb8%7C0%7C0%7C638249202319854798%7CUnknown%7CTWFpbGZsb3d8eyJWIjoiMC4wLjAwMDAiLCJQIjoiV2luMzIiLCJBTiI6Ik1haWwiLCJXVCI6Mn0%3D%7C3000%7C%7C%7C&sdata=mt1BLBfEcGPojHZWPuc%2Bco9M87TRbED%2BrZ2hoZZc9OY%3D&reserved=0. The Supporting Information is available free of charge at https://pubs.acs.org/doi/10.1021/acs.jproteome.3c00324.

Targeted proteomic panel with protein detectability and quantifiability, baseline characteristics of the overall British Columbia Post COVID-19—Interdisciplinary Clinical Care Network (PC-ICCN) patients who had been previously hospitalized for acute COVID-19,^55^ and six protein clusters identified along with the proteins in each cluster with functional analyses included in Figures 2 and 3 (PDF)
