# Supplementary material for: Proteomic Evolution from Acute to Post-COVID-19 Conditions
Source: J Proteome Res. 2023 Dec 4;23(1):52–70. doi: 10.1021/acs.jproteome.3c00324 (PMC10775146; doi:10.1021/acs.jproteome.3c00324)
Supplement: Supplementary file 1 — pr3c00324_si_001.pdf [file pr3c00324_si_001.pdf]

Supporting Information

Proteomic Evolution from Acute to Post-COVID-19 Conditions

Yassene Mohammed [1,2,3*], Karen Tran[4], Chris Carlsten[5], Christopher Ryerson[5], Alyson Wong[5], Terry Lee[6], Matthew P. Cheng[7], Donald C. Vinh[7], Todd C. Lee[7], Brent W. Winston[8], David Sweet[9], John H. Boyd[10,11], Keith R. Walley[10,11], Greg Haljan[12], Allison McGeer[13], Francois Lamontagne[14], Robert Fowler[15], David Maslove[16], Joel Singer[6], David M. Patrick[17], John C. Marshall[18], Srinivas Murthy[19], Fagun Jain[20], Christoph H. Borchers[21-24], David R. Goodlett[2], Adeera Levin[25], James A. Russell[10,11] and ARBs CORONA I.

Author affiliations:

1.Center for Proteomics and Metabolomics, Leiden University Medical Center, Leiden 2333 ZA, The Netherlands.

2. UVic-Genome BC Proteomics Centre, University of Victoria, Victoria V8Z 5N3, BC, Canada

3. Gerald Bronfman Department of Oncology, McGill University, Montreal, QC H3A 0G4, Canada

4. Division of General Internal Medicine, Vancouver General Hospital and University of British Columbia, 2775 Laurel St, Vancouver, BC V5Z 1M9, Canada.

5. Division of Respiratory Medicine, Vancouver General Hospital, University of British Columbia, Vancouver, BC V5Z 1M9, Canada.

6. Centre for Health Evaluation and Outcome Science (CHEOS), St. Paul's Hospital, University of British Columbia, 1081 Burrard Street, Vancouver, BC V6Z 1Y6, Canada.

7. Division of Infectious Diseases, Department of Medicine, McGill University Health Centre, Montreal, PQ H4A 3J1, Canada.

8. Departments of Critical Care Medicine, Medicine and Biochemistry and Molecular Biology, Foothills Medical Centre and University of Calgary, 1403 29 Street NW, Calgary, Alberta T2N 4N1, Canada.

9. Division of Critical Care Medicine, Vancouver General Hospital, 2775 Laurel St, Vancouver, BC V5Z 1M9, Canada.

10. Centre for Heart Lung Innovation, St. Paul’s Hospital, University of British Columbia, 1081 Burrard Street, Vancouver, BC V6Z 1Y6, Canada.
11. Division of Critical Care Medicine, St. Paul’s Hospital, University of British Columbia, 1081 Burrard Street, Vancouver, BC V6Z 1Y6, Canada.
12. Department of Medicine, Surrey Memorial Hospital, 13750 96th Avenue, Surrey, BC V3V 1Z2, Canada.
13. Mt. Sinai Hospital and University of Toronto, 600 University Avenue, Toronto, ON M5G 1X5, Canada.
14. University of Sherbrooke, Sherbrooke, PQ J1K 2R1, Canada.
15. Sunnybrook Health Sciences Centre, 2075 Bayview Avenue, Toronto, ON M4N 3M5, Canada.
16. Department of Critical Care, Kingston General Hospital and Queen's University, 76 Stuart Street, Kingston, ON K7L 2V7, Canada.
17. British Columbia Centre for Disease Control (BCCDC) and University of British Columbia, 655 West 12th Avenue, Vancouver, BC V5Z 4R4, Canada.
18. Department of Surgery, St. Michael’s Hospital, 30 Bond Street, Toronto, ON M5B 1W8, Canada.
19. BC Children’s Hospital and University of British Columbia, 4500 Oak Street, Vancouver, BC V6H 3N1, Canada.
20. Black Tusk Research Group, Vancouver, BC V6Z 2C7, Canada.
21. Segal Cancer Proteomics Centre, Lady Davis Institute for Medical Research, Jewish General Hospital, McGill University, Montreal, QC H3T 1E2, Canada.
22. Gerald Bronfman Department of Oncology, Jewish General Hospital, Montreal, QC H3T 1E2, Canada.
23. Division of Experimental Medicine, McGill University, Montreal, QC H3T 1E2, Canada.
24. Department of Pathology, McGill University, Montreal, QC H3T 1E2, Canada.
25. Division of Nephrology, St. Paul’s Hospital, 1081 Burrard Street, Vancouver, BC V6Z 1Y6, Canada.

***Corresponding author:**
Yassene Mohammed
Center for Proteomics and Metabolomics

Leiden University Medical Center, Leiden, the Netherlands
E-mail: y.mohammed@lumc.nl

Keywords: COVID-19, Post-COVID-19 condition, restrictive lung disease, targeted quantitative proteomics

Table S1. Targeted proteomics panel with protein detectability and quantifiability.

Protein	Gene	Protein name	Surrogate peptide	LLOQ	Mean of all measurements	Detected	Measurements above LLOQ	Quantified (min 1% of measurements above LLOQ)	Measurements above 50% LLOQ	Quantified (min 1% of measurements above 50% LLOQ)	Mean above 50% LLOQ
**P10809**	HSPD1	60 kDa heat shock protein, mitochondrial	GIIDPTK	4.61	1.11	Yes	0	No	5	Yes	No
**P08253**	MMP2	72 KDa type IV collagenase	IDAVYEAPQEEK	6.79	2.36	Yes	0	No	22	Yes	No
**P11021**	HSPA5	78 kDa glucose-regulated protein	ITPSYVAFTPEGER	24.93	41.68	Yes	184	Yes	191	Yes	Yes
**O95450**	ADAMTS2	A disintegrin and metalloproteinase with thrombospondin motifs 2	IILLSYGK	1.68	0.53	Yes	0	No	19	Yes	No
**P59510**	ADAMTS20	A disintegrin and metalloproteinase with thrombospondin motifs 20	IPAGATNVDIR	10.5	3.45	Yes	0	No	3	Yes	No
**Q9P2N4**	ADAMTS9	A disintegrin and metalloproteinase with thrombospondin motifs 9	LYNPDVR	17.16	2.41	Yes	0	No	0	No	No
**P62736**	ACTA2	Actin, aortic smooth muscle	SYELPDGQVITIGNER	31.65	214.71	Yes	185	Yes	191	Yes	Yes
**Q8IZF2**	ADGRF5	Adhesion G protein-coupled receptor F5	DVIVHPLPLK	0.56	2.52	Yes	191	Yes	191	Yes	Yes
**Q9HDC9**	APMAP	Adipocyte plasma membrane-associated protein	LLEYDTVTR	5.53	17.97	Yes	191	Yes	191	Yes	Yes
**Q15848**	ADIPOQ	Adiponectin	IFYNQQNHYDGSTGK	6.62	76.58	Yes	191	Yes	191	Yes	Yes
**P35318**	ADM	ADM	LDVASEFR	14.22	2.73	Yes	0	No	10	Yes	No
**P43652**	AFM	Afamin	DADPDTFFAK	58.24	356.72	Yes	191	Yes	191	Yes	Yes
**P02763**	ORM1	Alpha-1-acid glycoprotein 1	NWGLSVYADKPETTK	111.25	8033.4	Yes	191	Yes	191	Yes	Yes
**P01011**	SERPINA3	Alpha-1-antichymotrypsin	EIGELYLPK	28.27	4765.71	Yes	191	Yes	191	Yes	Yes
**P01009**	SERPINA1	Alpha-1-antitrypsin	SVLGQLGITK	50.95	21358.73	Yes	191	Yes	191	Yes	Yes
**P04217**	A1BG	Alpha-1B-glycoprotein	LETPDFQLFK	60.43	1956.85	Yes	189	Yes	189	Yes	Yes
**P08697**	SERPINF2	Alpha-2-antiplasmin	LGNQEPGGQTALK	89.83	820.93	Yes	191	Yes	191	Yes	Yes
**P02765**	AHSG	Alpha-2-HS-glycoprotein	FSVVYAK	32.22	3429.45	Yes	191	Yes	191	Yes	Yes
**P01023**	A2M	Alpha-2-macroglobulin	AIGYLNTGYQR	53.15	7804.43	Yes	191	Yes	191	Yes	Yes
**P03950**	ANG	Angiogenin	DINTFIHGNK	4.27	0.91	Yes	0	No	0	No	No
**Q9Y5C1**	ANGPTL3	Angiopoietin-related protein 3	DLVFSTWDHK	67.74	14.35	Yes	0	No	0	No	No
**P01019**	AGT	Angiotensinogen	ALQDQLVLVAAK	63.26	693.95	Yes	191	Yes	191	Yes	Yes
**P01008**	SERPINC1	Antithrombin-III	DDLYVSDAFHK	177.95	23693.71	Yes	191	Yes	191	Yes	Yes
**P02647**	APOA1	Apolipoprotein A-I	ATEHLSTLSEK	525.57	32177.83	Yes	191	Yes	191	Yes	Yes
**P02652**	APOA2	Apolipoprotein A-II	SPELQAEAK	102.18	11959.12	Yes	191	Yes	191	Yes	Yes
**P06727**	APOA4	Apolipoprotein A-IV	LGEVNTYAGDLQK	82.75	1096.54	Yes	191	Yes	191	Yes	Yes
**P04114**	APOB	Apolipoprotein B-100	FPEVDVLTK	7.29	209.71	Yes	191	Yes	191	Yes	Yes
**P02654**	APOC1	Apolipoprotein C-I	EWFSETFQK	61.6	2808.41	Yes	191	Yes	191	Yes	Yes
**P02655**	APOC2	Apolipoprotein C-II	TYLPAVDEK	9.01	1627.78	Yes	191	Yes	191	Yes	Yes
**P02656**	APOC3	Apolipoprotein C-III	GWVTDGFSSLK	55.18	6089.94	Yes	191	Yes	191	Yes	Yes
**P55056**	APOC4	Apolipoprotein C-IV	ELLETVVNR	10.73	53.08	Yes	176	Yes	187	Yes	Yes
**P05090**	APOD	Apolipoprotein D	NILTSNNIDVK	42.95	2010.58	Yes	191	Yes	191	Yes	Yes
**P02649**	APOE	Apolipoprotein E	LGPLVEQGR	12.63	727.24	Yes	191	Yes	191	Yes	Yes
**Q13790**	APOF	Apolipoprotein F	SGVQQLIQYYQDQK	16.21	328.99	Yes	191	Yes	191	Yes	Yes
**O14791**	APOL1	Apolipoprotein L1	VAQELEEK	24.38	383.42	Yes	191	Yes	191	Yes	Yes
**O95445**	APOM	Apolipoprotein M	AFLLTPR	7.99	315.57	Yes	191	Yes	191	Yes	Yes
**P08519**	LPA	Apolipoprotein(a)	GTYSTTVTGR	64.3	316.58	Yes	159	Yes	185	Yes	Yes
**P11511**	CYP19A1	Aromatase	NMLEMIFTPR	53.57	15.48	Yes	0	No	0	No	No
**P16066**	NPR1	Atrial natriuretic peptide receptor 1	ITDYGLESFR	6.57	0.61	Yes	0	No	0	No	No
**O75882**	ATRN	Attractin	SVNNVVVR	9.49	80.59	Yes	191	Yes	191	Yes	Yes
**Q8WXX7**	AUTS2	Autism susceptibility gene 2 protein	ALSLASSSGSDK	16.14	5.27	Yes	0	No	0	No	No
**Q8NDB2**	BANK1	B-cell scaffold protein with ankyrin repeats	LTIVHHPGGK	28.45	6.17	Yes	0	No	0	No	No
**P02749**	APOH	Beta-2-glycoprotein 1	ATVVYQGER	73.8	2501.41	Yes	191	Yes	191	Yes	Yes
**P61769**	B2M	Beta-2-microglobulin	VNHVTLSQPK	19.74	165.72	Yes	191	Yes	191	Yes	Yes
**Q96KN2**	CNDP1	Beta-Ala-His dipeptidase	ALEQDLPVNIK	16.23	82.62	Yes	191	Yes	191	Yes	Yes
**P01138**	NGF	Beta-nerve growth factor	TTATDIK	17.93	3.66	Yes	1	No	19	Yes	No
**P43251**	BTD	Biotinidase	SHLIIAQVAK	4.23	83.99	Yes	191	Yes	191	Yes	Yes
**P04003**	C4BPA	C4b-binding protein alpha chain	EDVYVVGTVLR	30.39	2437.38	Yes	191	Yes	191	Yes	Yes
**P55290**	CDH13	Cadherin-13	INENTGSVSVTR	55.66	-7.51	No	0	No	0	No	No
**P33151**	CDH5	Cadherin-5	ELDSTGTPTGK	24.95	32.59	Yes	128	Yes	187	Yes	Yes

**P06881**	CALCA	Calcitonin gene-related peptide 1	NNFVPTNVGSK	4.38	0.71	Yes	0	No	1	No	No
**P01258**	CALCA1	Calcitonin	FHTFPQTAIGVGAPGK	7.38	3.91	Yes	0	No	109	Yes	Yes
**P51911**	CNN1	Calponin-1	VNVGVK	12.25	0	No	0	No	0	No	No
**P00915**	CA1	Carbonic anhydrase 1	VLDALQAIK	6.07	150.16	Yes	186	Yes	191	Yes	Yes
**Q96IY4**	CPB2	Carboxypeptidase B2	IAWHVIR	38.96	68.83	Yes	183	Yes	191	Yes	Yes
**P15169**	CPN1	Carboxypeptidase N catalytic chain	SIPQVSPVR	2	99.46	Yes	191	Yes	191	Yes	Yes
**P22792**	CPN2	Carboxypeptidase N subunit 2	AGGSWDLAVQER	136.97	176.18	Yes	133	Yes	188	Yes	Yes
**Q9NQ79**	CRTAC1	Cartilage acidic protein 1	GVASLFAGR	24.39	16.97	Yes	24	Yes	140	Yes	Yes
**P49913**	CAMP	Cathelicidin antimicrobial peptide	AIDGINQR	14.62	15.95	Yes	82	Yes	179	Yes	Yes
**P11717**	IGF2R	Cation-independent mannose-6-phosphate receptor	GHQAFDVGQPR	32.72	10.39	Yes	0	No	9	Yes	No
**P29965**	CD40LG	CD40 ligand	SQFEGFVK	58.79	3.11	Yes	0	No	0	No	No
**P16070**	CD44	CD44 antigen	YGFIEGHVVIPR	39.4	20.67	Yes	4	Yes	92	Yes	Yes
**O43866**	CD5L	CD5 antigen-like	LVGGLHR	39.76	322.13	Yes	191	Yes	191	Yes	Yes
**P00450**	CP	Ceruloplasmin	IYHSHIDAPK	42.74	1752.01	Yes	191	Yes	191	Yes	Yes
**P11597**	CETP	Cholesteryl ester transfer protein	GVSLFDIINPEIITR	55.48	19.36	Yes	0	No	5	Yes	No
**P06276**	BCHE	Cholinesterase	YLTLNTESTR	6.42	40.99	Yes	191	Yes	191	Yes	Yes
**P10645**	CHGA	Chromogranin-A	ELQDLALQGAK	27.34	6.65	Yes	0	No	8	Yes	No
**O00501**	CLDN5	Claudin-5	PDLSFPVK	7.22	-1.42	No	0	No	0	No	No
**P10909**	CLU	Clusterin	ELDESLQVAER	24.59	2087.44	Yes	191	Yes	191	Yes	Yes
**P00740**	F9	Coagulation factor IX	SALVLQYLR	51.35	41.22	Yes	28	Yes	183	Yes	Yes
**P12259**	F5	Coagulation factor V	AEVDDVIQVR	71.58	39.3	Yes	1	No	115	Yes	Yes
**P08709**	F7	Coagulation factor VII	VSQYIEWLQK	10.49	6.31	Yes	8	Yes	121	Yes	Yes
**P00451**	F8	Coagulation factor VIII	LHPTHYSIR	81.81	0	No	0	No	0	No	No
**P00742**	F10	Coagulation factor X	MLEVPYVDR	15.04	111.78	Yes	191	Yes	191	Yes	Yes
**P03951**	F11	Coagulation factor XI	TSESGLPSTR	39.55	58.27	Yes	167	Yes	191	Yes	Yes
**P00748**	F12	Coagulation factor XII	EQPPSLTR	8.17	282.16	Yes	191	Yes	191	Yes	Yes
**P00488**	F13A1	Coagulation factor XIII A chain	GTYIPVPIVSELQSGK	9.09	54.68	Yes	191	Yes	191	Yes	Yes
**P05160**	F13B	Coagulation factor XIII B chain	IQTHSTTYR	38.37	99.67	Yes	186	Yes	191	Yes	Yes
**P02452**	COL1A1	Collagen alpha-1(I) chain	GVVGLPGQR	14.24	2.25	Yes	0	No	0	No	No
**P02461**	COL3A1	Collagen alpha-1(III) chain	GGAGPPGPEGGK	29.75	10.66	Yes	0	No	0	No	No
**P39060**	COL18A1	Collagen alpha-1(XVIII) chain	AVGLAGTFR	11.71	6.2	Yes	5	Yes	88	Yes	Yes
**P08123**	COL1A2	Collagen alpha-2(I) chain	GVVGPQGAR	3.46	0.86	Yes	0	No	7	Yes	No
**P02745**	C1QA	Complement C1q subcomponent subunit A	PAFSAIR	14.82	155.61	Yes	191	Yes	191	Yes	Yes
**P02746**	C1QB	Complement C1q subcomponent subunit B	IAFSATR	5.38	270.25	Yes	191	Yes	191	Yes	Yes
**P02747**	C1QC	Complement C1q subcomponent subunit C	FQSVFTVTR	7.04	542.05	Yes	191	Yes	191	Yes	Yes
**P00736**	C1R	Complement C1r subcomponent	GLTLHLK	6.28	356.55	Yes	191	Yes	191	Yes	Yes
**Q9NZP8**	C1RL	Complement C1r subcomponent-like protein	VVVHPDYR	9.18	57.11	Yes	191	Yes	191	Yes	Yes
**P09871**	C1S	Complement C1s subcomponent	TNFDNDIALVR	53.03	332.59	Yes	191	Yes	191	Yes	Yes
**P06681**	C2	Complement C2	HAFILQDTK	12.46	120.95	Yes	191	Yes	191	Yes	Yes
**P01024**	C3	Complement C3	TGLQEVEVK	61.43	4724.49	Yes	191	Yes	191	Yes	Yes
**P01031**	C5	Complement C5	VFQFLEK	7.57	207.97	Yes	191	Yes	191	Yes	Yes
**P13671**	C6	Complement component C6	DLHLSDVFLK	118.32	165.64	Yes	147	Yes	185	Yes	Yes
**P10643**	C7	Complement component C7	AASGTQNNVLR	55.51	192.54	Yes	191	Yes	191	Yes	Yes
**P07357**	C8A	Complement component C8 alpha chain	MESLGITSR	12.48	255.53	Yes	191	Yes	191	Yes	Yes
**P07358**	C8B	Complement component C8 beta chain	SDLEVAHYK	31.93	138.14	Yes	191	Yes	191	Yes	Yes
**P02748**	C9	Complement component C9	LSPIYNLVPVK	60.16	540.63	Yes	191	Yes	191	Yes	Yes
**P00751**	CFB	Complement factor B	EELLPAQDIK	12.69	1646.55	Yes	191	Yes	191	Yes	Yes
**P00746**	CFD	Complement factor D	THHDGAITER	17.45	54.57	Yes	191	Yes	191	Yes	Yes
**P08603**	CFH	Complement factor H	SSQESYAHGTK	31.98	1804.54	Yes	191	Yes	191	Yes	Yes
**P05156**	CFI	Complement factor I	VFSLQWGEVK	129.07	339.35	Yes	191	Yes	191	Yes	Yes
**P0C0L5**	PTK2	Focal adhesion kinase 1	VGDTLNLNLR	9.85	2459.76	Yes	191	Yes	191	Yes	Yes
**P0C0L4**	C4A	Complement C4-A	VLSLAQEQVGGSPEK	71.28	1873.44	Yes	191	Yes	191	Yes	Yes
**P08185**	SERPINA6	Corticosteroid-binding globulin	WSAGLTSSQVDLYIPK	427.3	345	Yes	33	Yes	182	Yes	Yes
**P12277**	CKB	Creatine kinase B-type	DLFDPIIEDR	13.42	3.35	Yes	0	No	0	No	No
**P02741**	CRP	C-reactive protein	AFVFPK	16.68	806.43	Yes	174	Yes	190	Yes	Yes
**P06732**	CKM	Creatine kinase M-type	FEEILTR	7	2.72	Yes	9	Yes	36	Yes	No
**P01034**	CST3	Cystatin-C	ALDFAVGEYNK	10.33	43.75	Yes	191	Yes	191	Yes	Yes
**P15924**	DSP	Desmoplakin	AELIVQPELK	9.28	3.58	Yes	0	No	10	Yes	No
**O94907**	DKK1	Dickkopf-related protein 1 and 2	GSHGLEIFQR	24.45	3.86	Yes	0	No	0	No	No
**Q01459**	CTBS	Di-N-acetylchitobiase	ATYIQNYR	50.97	16.34	Yes	0	No	7	Yes	No
**P15502**	ELN	Elastin	LPGGYGLPYTTGK	16.06	6.06	Yes	0	No	0	No	No
**Q9Y5X9**	LIPG	Endothelial lipase	LVSALHTR	1.39	0.57	Yes	0	No	24	Yes	No

**Q9UNN8**	PROCR	Endothelial protein C receptor	TLAFPLTIR	18.51	10.94	Yes	6	Yes	129	Yes	Yes
**P00533**	EGFR	Epidermal growth factor receptor	IPLENLQIIR	4.41	1.14	Yes	0	No	0	No	No
**P16581**	SELE	E-selectin	YTHLVAIQNK	19.93	8.33	Yes	1	No	33	Yes	No
**Q16610**	ECM1	Extracellular matrix protein 1	NVALVSGDTENAK	9.96	58.51	Yes	191	Yes	191	Yes	Yes
**P05413**	FABP3	Fatty acid-binding protein, heart	SLGVGFATR	18.25	7.03	Yes	0	No	1	No	No
**P02794**	FTH1	Ferritin heavy chain	NVNQSLLELHK	1.22	0.59	Yes	2	Yes	33	Yes	No
**P02792**	FTL	Ferritin light chain	LGGPEAGLGEYLFER	12.77	-0.35	No	0	No	0	No	No
**Q9UGM5**	FETUB	Fetuin-B	LVVLPFPK	56.42	47.6	Yes	47	Yes	179	Yes	Yes
**P02671**	FGA	Fibrinogen alpha chain	VQHIQLLQK	92.86	16151.61	Yes	191	Yes	191	Yes	Yes
**P02675**	FGB	Fibrinogen beta chain	HQLYIDETVNSNIPTNLR	47.34	18395.82	Yes	190	Yes	191	Yes	Yes
**P02679**	FGG	Fibrinogen gamma chain	YEASILTHDSSIR	73.62	10961.27	Yes	190	Yes	190	Yes	Yes
**P02751**	FN1	Fibronectin	HTSVQTTSSGSGPFTDVR	46.07	268.26	Yes	186	Yes	190	Yes	Yes
**P23142**	FBLN1	Fibulin-1	TGYYFDGISR	7.42	125.72	Yes	191	Yes	191	Yes	Yes
**Q15485**	FCN2	Ficolin-2	GTHGSFANGINWK	145.3	55.75	Yes	0	No	13	Yes	No
**O75636**	FCN3	Ficolin-3	YAVSEAAAHK	442.85	370.52	Yes	27	Yes	191	Yes	Yes
**Q12841**	FSTL1	Follistatin-related protein 1	YVQELQK	26.88	13	Yes	0	No	70	Yes	No
**P05062**	ALDOB	Fructose-bisphosphate aldolase B	ALQASALAAWGGK	36.19	14.25	Yes	4	Yes	24	Yes	No
**P17931**	LGALS3	Galectin-3	IALDFQR	2.93	1.43	Yes	8	Yes	84	Yes	No
**Q08380**	LGALS3BP	Galectin-3-binding protein	SDLAVPSELALLK	59.59	46.1	Yes	24	Yes	100	Yes	Yes
**P09104**	ENO2	Gamma-enolase	YITGDQLGALYQDFVR	12.68	1.37	Yes	0	No	0	No	No
**P06396**	GSN	Gelsolin	AGALNSNDAFVLK	156.17	4016.71	Yes	191	Yes	191	Yes	Yes
**P14136**	GFAP	Glial fibrillary acidic protein	LADVYQAELR	6.29	0.77	Yes	0	No	35	Yes	No
**Q12879**	GRIN2A	Glutamate receptor ionotropic, NMDA 2A	FSYIPEAK	23.54	7.04	Yes	0	No	0	No	No
**Q13224**	GRIN2B	Glutamate receptor ionotropic, NMDA 2B	EPGGPSFTIGK	39.45	13.16	Yes	0	No	0	No	No
**P22352**	GPX3	Glutathione peroxidase 3	QEPGENSEILPTLK	10.47	138.38	Yes	191	Yes	191	Yes	Yes
**P09211**	GSTP1	Glutathione S-transferase P	TLGLYGK	692.49	-136.88	No	0	No	0	No	No
**P00738**	HP	Haptoglobin	DIAPTLTLYVGK	378.27	32927.7	Yes	191	Yes	191	Yes	Yes
**P04792**	HSPB1	Heat shock protein beta-1	LFDQAFGLPR	67.38	13.9	Yes	0	No	0	No	No
**P02790**	HPX	Hemopexin	NFPSPVDAAFR	61.72	9629.27	Yes	191	Yes	191	Yes	Yes
**P05546**	SERPIND1	Heparin cofactor 2	TLEAQLTPR	13.54	777.28	Yes	191	Yes	191	Yes	Yes
**P26927**	MST1	Hepatocyte growth factor-like protein	SPLNDFQVLR	49.31	15.59	Yes	0	No	3	Yes	No
**P04196**	HRG	Histidine-rich glycoprotein	ADLFYDVEALDLESPK	29.62	949.08	Yes	191	Yes	191	Yes	Yes
**Q86YZ3**	HRNR	Hornerin	GSGSGQSPSSGQHGTGFGR	145.58	46.29	Yes	0	No	0	No	No
**Q14520**	HABP2	Hyaluronan-binding protein 2	VVLGDQDLK	4.89	160.5	Yes	191	Yes	191	Yes	Yes
**P01857**	IGHG1	Immunoglobulin heavy constant gamma 1	GPSVFPLAPSSK	51.31	14862.59	Yes	191	Yes	191	Yes	Yes
**P04220**	IGHM	Immunoglobulin heavy constant mu	VSVFVPPR	42.71	4202.77	Yes	191	Yes	191	Yes	Yes
**Q9Y6R7**	FCGBP	IgGFc-binding protein	GATTSPGVYELSSR	9.85	14.36	Yes	161	Yes	191	Yes	Yes
**P69905**	HBA2	Hemoglobin subunit alpha	VGAHAGEYGAEALER	35.63	2982.6	Yes	191	Yes	191	Yes	Yes
**P01625**	IGKV4-1	Immunoglobulin kappa variable 4-1	NYLAWYQQKPGQPPK	8.98	1310.22	Yes	191	Yes	191	Yes	Yes
**P05019**	IGF1	Insulin-like growth factor I	GFYFNKPTGYGSSSR	47.41	22.8	Yes	1	No	67	Yes	No
**P08833**	IGFBP1	Insulin-like growth factor-binding protein 1	ALPGEQQPLHALTR	14.03	2.35	Yes	1	No	3	Yes	No
**P18065**	IGFBP2	Insulin-like growth factor-binding protein 2	LIQGAPTIR	4.19	8.97	Yes	143	Yes	189	Yes	Yes
**P17936**	IGFBP3	Insulin-like growth factor-binding protein 3	FLNVLSPR	2.76	22.49	Yes	190	Yes	191	Yes	Yes
**P35858**	IGFALS	Insulin-like growth factor-binding protein complex acid labile subunit	NLIAAVAPGAFLGLK	18.89	85.18	Yes	189	Yes	190	Yes	Yes
**P19827**	ITIH1	Inter-alpha-trypsin inhibitor heavy chain H1	GSLVQASEANLQAAQDFVR	649.61	2555.57	Yes	189	Yes	191	Yes	Yes
**P19823**	ITIH2	Inter-alpha-trypsin inhibitor heavy chain H2	SLAPTAAAK	25.82	1132.18	Yes	191	Yes	191	Yes	Yes
**Q14624**	ITIH4	Inter-alpha-trypsin inhibitor heavy chain H4	SPEQQETVLDGNLIIR	54.07	1274.74	Yes	191	Yes	191	Yes	Yes
**P05362**	ICAM1	Intercellular adhesion molecule 1	LLGIETPLPK	9.23	5.95	Yes	10	Yes	149	Yes	Yes
**P22301**	IL10	Interleukin-10	AHVNSLGENLK	7.28	2.56	Yes	0	No	25	Yes	No
**P05231**	IL6	Interleukin-6	FESSEEQAR	36.78	12.7	Yes	0	No	0	No	No
**P03956**	MMP1	Interstitial collagenase	AFQLWSNVTPLTFTK	5.51	1.36	Yes	0	No	0	No	No
**P29622**	SERPINA4	Kallistatin	VGSALFLSHNLK	42.77	57.05	Yes	121	Yes	172	Yes	Yes
**P13645**	KRT10	Keratin, type I cytoskeletal 10	SLLEGEGSSGGGGR	84.72	16.18	Yes	0	No	0	No	No
**P35527**	KRT9	Keratin, type I cytoskeletal 9	TLLDIDNTR	12.03	0.52	Yes	1	No	1	No	No
**P35908**	KRT2	Keratin, type II cytoskeletal 2 epidermal	YEELQVTVGR	294.1	7.32	Yes	0	No	0	No	No
**P01042**	KNG1	Kininogen-1	DIPTNSPELEETLTHTITK	39.4	1490.94	Yes	191	Yes	191	Yes	Yes
**P02788**	LTF	Lactotransferrin	YLGPQYVAGITNLK	22.51	10.9	Yes	1	No	55	Yes	No
**P02750**	LRG1	Leucine-rich alpha-2-glycoprotein	DLLLPQPDLR	56.24	460.4	Yes	191	Yes	191	Yes	Yes
**P18428**	LBP	Lipopolysaccharide-binding protein	ITLPDFTGDLR	69.2	147.01	Yes	131	Yes	191	Yes	Yes

**P14151**	SELL	L-selectin	AEIEYLEK	5.25	31.86	Yes	191	Yes	191	Yes	Yes
**P51884**	LUM	Lumican	SLEDLQLTHNK	55.89	361.56	Yes	191	Yes	191	Yes	Yes
**P61626**	LYZ	Lysozyme C	AWVAWR	4.5	64.25	Yes	191	Yes	191	Yes	Yes
**P48740**	MASP1	Mannan-binding lectin serine protease 1	TGVITSPDFPNPYPK	144.48	105.01	Yes	0	No	188	Yes	Yes
**O00187**	MASP2	Mannan-binding lectin serine protease 2	WPEPVFGR	53.96	19.3	Yes	0	No	12	Yes	No
**P11226**	MBL2	Mannose-binding protein C	WLTFSLGK	46.5	23.58	Yes	11	Yes	68	Yes	Yes
**P08493**	MGP	Matrix Gla protein	NANTFISPQQR	48.56	-7.29	No	0	No	0	No	No
**P14780**	MMP9	Matrix metalloproteinase-9	AVIDDAFAR	7.96	2.97	Yes	5	Yes	30	Yes	No
**P08582**	MELTF	Melanotransferrin	YYDYSGAFR	26.89	3.63	Yes	0	No	0	No	No
**P01033**	TIMP1	Metalloproteinase inhibitor 1	GFQALGDAADIR	34.31	9.2	Yes	0	No	6	Yes	No
**P16035**	TIMP2	Metalloproteinase inhibitor 2	EYLIAGK	4.77	4.71	Yes	83	Yes	187	Yes	Yes
**Q99727**	TIMP4	Metalloproteinase inhibitor 4	VVPASADPADTEK	7.68	2.69	Yes	0	No	0	No	No
**P10636**	MAPT	Microtubule-associated protein tau	EADLPEPSEK	15	2.85	Yes	0	No	0	No	No
**Q8WXI7**	MUC16	Mucin-16	ELGPYTLDR	10.86	3.04	Yes	2	Yes	25	Yes	No
**P02686**	MBP	Myelin basic protein	GVDAQGTLSK	4.2	1.35	Yes	1	No	4	Yes	No
**P24158**	PRTN3	Myeloblastin	LVNVVLGAHNVR	67.03	5.16	Yes	2	Yes	2	Yes	No
**P05164**	MPO	Myeloperoxidase	VFFASWR	46.25	8.76	Yes	0	No	0	No	No
**O94760**	DDAH1	N(G),N(G)-dimethylarginine dimethylaminohydrolase 1	TPEEYPESAK	73.66	19.12	Yes	0	No	0	No	No
**Q96PD5**	PGLYRP2	N-acetylmuramoyl-L-alanine amidase	AGLLRPDYALLGHR	94.6	219.02	Yes	189	Yes	191	Yes	Yes
**P16860**	NPPB	Natriuretic peptides B	EVATEGIR	3.85	1.19	Yes	2	Yes	17	Yes	No
**O60462**	NRP2	Neuropilin-2	ALQVVR	7.56	57.61	Yes	191	Yes	191	Yes	Yes
**P80188**	LCN2	Neutrophil gelatinase-associated lipocalin	ITLYGR	4.14	4.48	Yes	89	Yes	190	Yes	Yes
**P15531**	NME1	Nucleoside diphosphate kinase A, B	PFFAGLVK	7.49	1.33	Yes	0	No	0	No	No
**Q16625**	OCLN	Occludin	SLQSELDEINK	17.06	6.55	Yes	0	No	2	Yes	No
**P10451**	SPP1	Osteopontin	GDSVVYGLR	14.61	2.62	Yes	0	No	0	No	No
**P78380**	OLR1	Oxidized low-density lipoprotein receptor 1	LEGQISAR	8.65	5.34	Yes	14	Yes	101	Yes	Yes
**Q13219**	PAPPA	Pappalysin-1	AYLDVNELK	3.16	1.68	Yes	0	No	106	Yes	Yes
**Q06830**	PRDX1	Peroxiredoxin-1	ADEGISFR	11.94	5.55	Yes	2	Yes	65	Yes	No
**P32119**	PRDX2	Peroxiredoxin-2	GLFIIDGK	12.96	36.37	Yes	145	Yes	183	Yes	Yes
**P04180**	LCAT	Phosphatidylcholine-sterol acyltransferase	SSGLVSNAPGVQIR	24.41	83.52	Yes	191	Yes	191	Yes	Yes
**P55058**	PLTP	Phospholipid transfer protein	AVEPQLQEEER	13.79	38.06	Yes	189	Yes	191	Yes	Yes
**P80108**	GPLD1	Phosphatidylinositol-glycan-specific phospholipase D	FGSSLITVR	4.58	51.24	Yes	191	Yes	191	Yes	Yes
**P36955**	SERPINF1	Pigment epithelium-derived factor	LQSLFDSPDFSK	56.34	266.22	Yes	191	Yes	191	Yes	Yes
**P05155**	SERPING1	Plasma protease C1 inhibitor	FQPTLLTLPR	30.3	2064.77	Yes	191	Yes	191	Yes	Yes
**P05154**	SERPINA5	Plasma serine protease inhibitor	GFQQLLQELNQPR	14	79.56	Yes	188	Yes	191	Yes	Yes
**P05121**	SERPINE1	Plasminogen activator inhibitor 1	VFQQVAQASK	28.63	13.09	Yes	2	Yes	71	Yes	No
**P00747**	PLG	Plasminogen	EAQLPVIENK	54.19	1403.45	Yes	191	Yes	191	Yes	Yes
**P13796**	LCP1	Plastin-2	ISFDEFIK	38.51	21.51	Yes	5	Yes	77	Yes	Yes
**P16284**	PECAM1	Platelet endothelial cell adhesion molecule	SELVTVTESFSTPK	10.9	3.16	Yes	0	No	32	Yes	No
**Q9HCN6**	GP6	Platelet glycoprotein VI	EGDPAPYK	28.68	7.51	Yes	0	No	9	Yes	No
**Q13093**	PLA2G7	Platelet-activating factor acetylhydrolase	GSVHQNFADFTFATGK	69.28	16.78	Yes	0	No	0	No	No
**P20742**	PZP	Pregnancy zone protein	ISEITNIVSK	19.96	50	Yes	96	Yes	132	Yes	Yes
**P01210**	PENK	Proenkephalin-A	ELLETGDNR	3.21	0.82	Yes	0	No	3	Yes	No
**P01236**	PRL	Prolactin	IDNYLK	91.66	9.37	Yes	0	No	0	No	No
**P02760**	AMBP	Protein AMBP	HHGPTITAK	81.77	563.34	Yes	191	Yes	191	Yes	Yes
**P80511**	S100A12	Protein S100-A12	GHFDTLSK	24.8	12.96	Yes	5	Yes	44	Yes	Yes
**P06702**	S100A9	Protein S100-A9	DLQNFLK	10.2	57.02	Yes	130	Yes	188	Yes	Yes
**P04271**	S100B	Protein S100-B	EQEVVDK	308.29	54.72	Yes	0	No	5	Yes	No
**Q9UK55**	SERPINA10	Protein Z-dependent protease inhibitor	ETSNFGFSLLR	51.51	37.8	Yes	28	Yes	169	Yes	Yes
**Q99497**	PARK7	Parkinson disease protein 7	ALVILAK	0.87	2.97	Yes	182	Yes	190	Yes	Yes
**Q92954**	PRG4	Proteoglycan 4	DQYYNIDVPSR	6.84	44.82	Yes	191	Yes	191	Yes	Yes
**P00734**	F2	Prothrombin	ELLESYIDGR	72.34	3235.01	Yes	191	Yes	191	Yes	Yes
**P16109**	SELP	P-selectin	TWTWVGTK	13.98	6.54	Yes	3	Yes	71	Yes	No
**Q9UJF2**	RASAL2	Ras GTPase-activating protein nGAP	ETQSTPQSAPQVR	35.08	9.1	Yes	0	No	0	No	No
**Q9HD89**	RETN	Resistin	IQEVAGSLIFR	5.52	1.5	Yes	0	No	4	Yes	No
**P02753**	RBP4	Retinol-binding protein 4	YWGVASFLQK	55.24	990.55	Yes	191	Yes	191	Yes	Yes
**P02787**	TF	Serotransferrin	DGAGDVAFVK	235.95	15249.94	Yes	191	Yes	191	Yes	Yes
**P02768**	ALB	Serum albumin	LVNEVTEFAK	1124.9	579528.4	Yes	191	Yes	191	Yes	Yes
**P0DJI8**	SAA1	Serum amyloid A-1 and A-2 proteins	EANYIGSDK	10.24	3222.84	Yes	188	Yes	191	Yes	Yes
**P35542**	SAA4	Serum amyloid A-4 protein	GNYDAAQR	224.35	1318.9	Yes	191	Yes	191	Yes	Yes
**P02743**	APCS	Serum amyloid P-component	IVLGQEQDSYGGK	21.15	739.79	Yes	191	Yes	191	Yes	Yes

**P27169**	PON1	Serum paraoxonase/arylesterase 1	IFFYDSENPPASEVLR	48.23	760.58	Yes	191	Yes	191	Yes	Yes
**Q15166**	PON3	Serum paraoxonase/lactonase 3	ILIGTVFHK	8.27	4.45	Yes	10	Yes	104	Yes	Yes
**P04278**	SHBG	Sex hormone-binding globulin	TSSSFEVR	7.94	30.41	Yes	187	Yes	191	Yes	Yes
**P09486**	SPARC	SPARC	LEAGDHPVELLAR	7.91	4.64	Yes	20	Yes	86	Yes	Yes
**Q9NWM0**	SMOX	Spermine oxidase	YYSTTHGALLSGQR	9.1	2.66	Yes	0	No	1	No	No
**Q8IVG5**	SAMD9L	Sterile alpha motif domain-containing protein 9-like	ENVLDEVANAK	35.85	6.84	Yes	0	No	0	No	No
**P08254**	MMP3	Stromelysin-1	TYFFVEDK	68.28	2.47	Yes	0	No	0	No	No
**Q7Z7G0**	ABI3BP	Target of Nesh-SH3	IYLSDSLTGK	9.3	7.57	Yes	31	Yes	183	Yes	Yes
**Q9BXI6**	TBC1D10A	TBC1 domain family member 10A	YLPGYYSEK	7.74	0.42	Yes	0	No	0	No	No
**P22105**	TNXB	Tenascin-X	ILISGLEPSTPYR	9.31	4.96	Yes	0	No	107	Yes	Yes
**P24821**	TNC	Tenascin	FTTDLDSPR	22.16	10.13	Yes	0	No	35	Yes	No
**P05452**	CLEC3B	Tetranectin	NWETEITAQPDGGK	30.8	153.72	Yes	191	Yes	191	Yes	Yes
**P07204**	THBD	Thrombomodulin	SSVAADVISLLLNGDGGVGR	3.62	1.35	Yes	0	No	3	Yes	No
**P07996**	THBS1	Thrombospondin-1	GTLLALER	10.28	25.26	Yes	153	Yes	185	Yes	Yes
**P35443**	THBS4	Thrombospondin-4	KPQDFLEELK	32.99	7.69	Yes	0	No	0	No	No
**P05543**	SERPINA7	Thyroxine-binding globulin	AVLHIGEK	4.88	245.28	Yes	191	Yes	191	Yes	Yes
**P01266**	TG	Thyroglobulin	FSPDDSAGASALLR	9.43	1.5	Yes	0	No	0	No	No
**P10646**	TFPI	Tissue factor pathway inhibitor	FYYNSVIGK	15.82	4.99	Yes	0	No	0	No	No
**P00750**	PLAT	Tissue-type plasminogen activator	VVPGEEEQK	68.16	-5.19	No	0	No	0	No	No
**P35716**	SOX11	Transcription factor SOX-11	AAQSGDYGGAGDDYVLGSLR	16.12	-0.05	No	0	No	0	No	No
**P02786**	TFRC	Transferrin receptor protein 1	GFVEPDHYVVVGAQR	28.97	12.92	Yes	0	No	40	Yes	No
**P02766**	TTR	Transthyretin	GSPAINVAVHVFR	30.77	335.26	Yes	191	Yes	191	Yes	Yes
**P19438**	TNFRSF1A	Tumor necrosis factor receptor superfamily member 1A	LGLSDHEIDR	8.27	2.01	Yes	0	No	2	Yes	No
**P20333**	TNFRSF1B	Tumor necrosis factor receptor superfamily member 1B	DEQVPFSK	8.28	-0.87	No	0	No	0	No	No
**P19320**	VCAM1	Vascular cell adhesion protein 1	NTVISVNPSTK	8.9	14.47	Yes	153	Yes	186	Yes	Yes
**P49765**	VEGFB	Vascular endothelial growth factor B	VVSWIDVYTR	5.74	0.8	Yes	0	No	0	No	No
**O43915**	VEGFD	Vascular endothelial growth factor D	DLIQHPK	95.55	48.98	Yes	0	No	62	Yes	Yes
**Q9NY84**	VNN3	Vascular non-inflammatory molecule 3	TETPVSK	125.41	0.47	Yes	0	No	0	No	No
**Q6EMK4**	VASN	Vasorin	YLQGSSVQLR	8.06	14.09	Yes	182	Yes	191	Yes	Yes
**P02774**	GC	Vitamin D-binding protein	VLEPTLK	51.53	2341.24	Yes	191	Yes	191	Yes	Yes
**P04070**	PROC	Vitamin K-dependent protein C	LGEYDLR	9.94	29.34	Yes	189	Yes	190	Yes	Yes
**P07225**	PROS1	Vitamin K-dependent protein S	VYFAGFPR	16.03	319.46	Yes	191	Yes	191	Yes	Yes
**P22891**	PROZ	Vitamin K-dependent protein Z	GLLSGWAR	44.59	22.41	Yes	4	Yes	93	Yes	Yes
**P04004**	VTN	Vitronectin	FEDGVLDPDYPR	318.9	2499.45	Yes	191	Yes	191	Yes	Yes
**P04275**	VWF	von Willebrand factor	ILAGPAGDSNVVK	22.48	18.07	Yes	33	Yes	159	Yes	Yes
**P12955**	PEPD	Xaa-Pro dipeptidase	AVYEAVLR	5.56	6.13	Yes	109	Yes	190	Yes	Yes
**P25311**	AZGP1	Zinc-alpha-2-glycoprotein	EIPAWVPFDPAAQITK	34.41	568.2	Yes	191	Yes	191	Yes	Yes

Table S2. Baseline characteristics of the overall British Columbia Post COVID-19 – Interdisciplinary Clinical Care Network (PC-ICCN) patients who had been previously hospitalized for acute COVID-19 [55].

	**PC-ICCN Previously Hospitalized Patients n (%) or mean±SD**
Total patients	548 (100)
Time since diagnosis (days)	111±52
Time since symptom onset (days)	115±60
Age (years)	57±14
Female sex	242 (44.2)
Intensive care unit stay	193 (35.2)

Figure S1. The six protein clusters identified along with the proteins in each cluster with functional analyses included in Figure 2 and Figure 3.

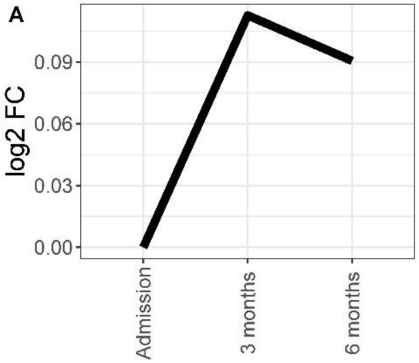

HSPA5	COL1A2	HPX	S100B
APOB	C1QA	FCGBP	PRG4
APOE	C1QC	IL10	F2
APOL1	C3	LYZ	SPARC
NGF	CFH	MASP1	TFRC
CALCA1	SERPINA6	NPPB	PROZ
CD5L	CTBS	SERPINE1	PEPD
CHGA	SELE	PLG	
F10	FCN2	PZP	
COL18A1	FCN3	AMBP	

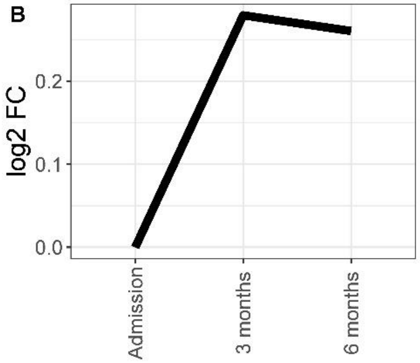

MMP2	CD44	SERPIND1	TNXB
ADIPOQ	CETP	IGF1	THBS1
A2M	CLU	KNG1	VASN
SERPINC1	F7	TIMP2	PROC
APOD	F12	LCAT	AZGP1
LPA	CFD	SERPINF1	
CNDP1	PROCR	GP6	
CDH5	ECM1	PARK7	
CPB2	FETUB	SHBG	
CAMP	FBLN1	ABI3BP	

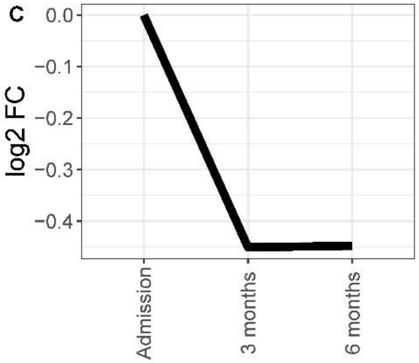

ADGRF5	C1RL	ALDOB	LBP	S100A9
APMAP	C2	LGALS3	MASP2	SERPINA10
ORM1	C6	LGALS3BP	MBL2	SAA1
SERPINA3	C9	GFAP	MMP9	SAA4
SERPINA1	CFB	GPX3	TIMP1	APCS
B2M	PTK2	HP	PRTN3	VCAM1
C4BPA	C4A	HBA2	PRDX2	VWF
CA1	CRP	IGFBP2	SERPING1	
CPN1	FGA	ITIH4	LCP1	
CP	FGB	LRG1	S100A12	

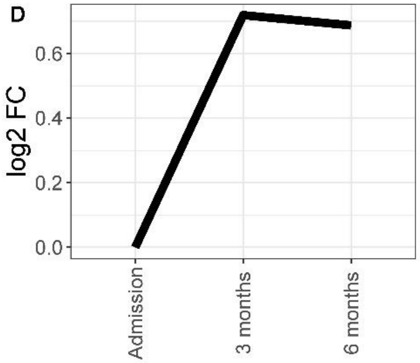

HSPD1	BTD	SELP
AHSG	CRTAC1	TF
APOA1	BCHE	ALB
APOA2	F13B	PON1
APOC2	GSN	PON3
APOC3	IGFALS	CLEC3B
APOC4	ITIH1	
APOM	ITIH2	
ATRN	PGLYRP2	
APOH	PLTP	

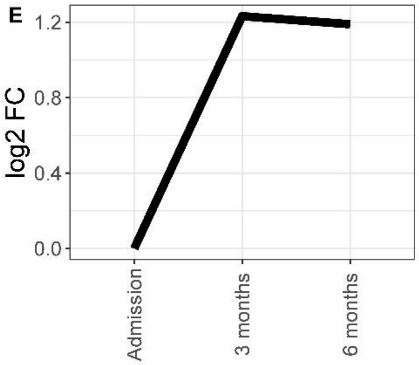

ACTA2	GPLD1
AFM	SERPINA5
APOA4	PECAM1
APOC1	RBP4
F13A1	TTR
FN1	
HRG	
IGFBP3	
SERPINA4	
LUM	

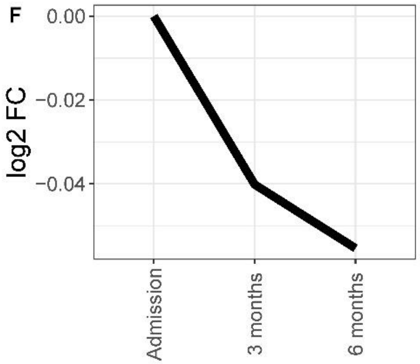

ADAMTS2	F5	CKM	IGHM	OCLN	VEGFD
ADAMTS20	F11	CST3	IGKV4.1	OLR1	GC
ADM	C1QB	DSP	IGFBP1	PAPPA	PROS1
A1BG	C1R	LIPG	ICAM1	PRDX1	VTN
SERPINF2	C1S	FTH1	LTF	PENK	
AGT	C5	FGG	SELL	RETN	
APOF	C7	FSTL1	MUC16	TNC	
CPN2	C8A	MST1	MBP	THBD	
IGF2R	C8B	HABP2	NRP2	SERPINA7	
F9	CFI	IGHG1	LCN2	TNFRSF1A	

Links

Full link Figure 2E

https://genemania.org/search/homo-sapiens/HSPA5/APOB/APOE/APOL1/NGF/CALCA1/CD5L/CHGA/F10/COL18A1/COL1A2/C1QA/C1QC/C3/CFH/SERPINA6/CTBS/SELE/FCN2/FCN3/HPX/FCGBP/IL10/LYZ/MASP1/NPPB/SERPINE1/PLG/PZP/AMBP/S100B/PRG4/F2/SPARC/TFRC/PROZ/PEPD/MMP2/ADIPOQ/A2M/SERPINC1/APOD/LPA/CNDP1/CDH5/CPB2/CAMP/CD44/CETP/CLU/F7/F12/CFD/PROCR/ECM1/FETUB/FBLN1/SERPIND1/IGF1/KNG1/TIMP2/LCAT/SERPINF1/GP6/PARK7/SHBG/ABI3BP/TNXB/THBS1/VASN/PROC/AZGP1/HSPD1/AHSG/APOA1/APOA2/APOC2/APOC3/APOC4/APOM/ATRN/APOH/BTD/CRTAC1/BCHE/F13B/GSN/IGFALS/ITIH1/ITIH2/PGLYRP2/PLTP/SELP/TF/ALB/PON1/PON3/CLEC3B/ACTA2/AFM/APOA4/APOC1/F13A1/FN1/HRG/IGFBP3/SERPINA4/LUM/GPLD1/SERPINA5/PECAM1/RBP4/TTR

Full link Figure 3C

https://genemania.org/search/homo-sapiens/ADGRF5/APMAP/ORM1/SERPINA3/SERPINA1/B2M/C4BPA/CA1/CPN1/CP/C1RL/C2/C6/C9/CFB/PTK2/C4A/CRP/FGA/FGB/ALDOB/LGALS3/LGALS3BP/GFAP/GPX3/HP/HBA2/IGFBP2/ITIH4/LRG1/LBP/MASP2/MBL2/MMP9/TIMP1/PRTN3/PRDX2/SERPING1/LCP1/S100A12/S100A9/SERPINA10/SAA1/SAA4/APCS/VCAM1/VWF/ADAMTS2/ADAMTS20/ADM/A1BG/SERPINF2/AGT/APOF/CPN2/IGF2R/F9/F5/F11/C1QB/C1R/C1S/C5/C7/C8A/C8B/CFI/CKM/CST3/DSP/LIPG/FTH1/FGG/FSTL1/MST1/HABP2/IGHG1/IGHM/IGKV4.1/IGFBP1/ICAM1/LTF/SELL/MUC16/MBP/NRP2/LCN2/OCLN/OLR1/PAPPA/PRDX1/PENK/RETN/TNC/THBD/SERPINA7/TNFRSF1A/VEGFD/GC/PROS1/VTN
